# A systematic review of randomised controlled trials assessing effectiveness of prosthetic and orthotic interventions

**DOI:** 10.1371/journal.pone.0192094

**Published:** 2018-03-14

**Authors:** Aoife Healy, Sybil Farmer, Anand Pandyan, Nachiappan Chockalingam

**Affiliations:** 1 School of Life Sciences and Education, Staffordshire University, Stoke On Trent, United Kingdom; 2 School of Health & Rehabilitation, Keele University, Keele, United Kingdom; Northwestern University, UNITED STATES

## Abstract

**Background:**

Assistive products are items which allow older people and people with disabilities to be able to live a healthy, productive and dignified life. It has been estimated that approximately 1.5% of the world’s population need a prosthesis or orthosis.

**Objective:**

The objective of this study was to systematically identify and review the evidence from randomized controlled trials assessing effectiveness and cost-effectiveness of prosthetic and orthotic interventions.

**Methods:**

Literature searches, completed in September 2015, were carried out in fourteen databases between years 1995 and 2015. The search results were independently screened by two reviewers. For the purpose of this manuscript, only randomized controlled trials which examined interventions using orthotic or prosthetic devices were selected for data extraction and synthesis.

**Results:**

A total of 342 randomised controlled trials were identified (319 English language and 23 non-English language). Only 4 of these randomised controlled trials examined prosthetic interventions and the rest examined orthotic interventions. These orthotic interventions were categorised based on the medical conditions/injuries of the participants. From these studies, this review focused on the medical condition/injuries with the highest number of randomised controlled trials (osteoarthritis, fracture, stroke, carpal tunnel syndrome, plantar fasciitis, anterior cruciate ligament, diabetic foot, rheumatoid and juvenile idiopathic arthritis, ankle sprain, cerebral palsy, lateral epicondylitis and low back pain). The included articles were assessed for risk of bias using the Cochrane Risk of Bias tool. Details of the clinical population examined, the type of orthotic/prosthetic intervention, the comparator/s and the outcome measures were extracted. Effect sizes and odds ratios were calculated for all outcome measures, where possible.

**Conclusions:**

At present, for prosthetic and orthotic interventions, the scientific literature does not provide sufficient high quality research to allow strong conclusions on their effectiveness and cost-effectiveness.

## Introduction

Assistive technology is a term normally used to cover the systems and services associated with assistive products and services [1]. Assistive products are items which allow people with disabilities to be able to live a healthy, productive and dignified life. In 2016 the WHO launched the Priority Assistive Products List [2] which identified fifty priority assistive products, this list is intended to promote access to these products. The importance of orthotics and prosthetics for people with disabilities was emphasised in this list with orthoses (lower and upper limb, and spinal), therapeutic footwear (diabetic, neuropathic and orthopaedic) and lower limb prostheses all named on the list.

The definitions for prosthesis and orthosis [3] as outlined within this manuscript are:

- Prosthesis (prosthetic device/product): externally applied device used to replace wholly, or in part, an absent or deficient limb segment (plural: prostheses). Common examples are transtibial and transfemoral prostheses for the lower limb and transradial or transhumeral prostheses for the upper limb.
- Orthosis (orthotic device/product): externally applied device used to modify the structural and functional characteristics of the neuromuscular and skeletal systems (plural: orthoses). Common examples are ankle braces, wrist splints, ankle foot orthoses, custom footwear and lumbosacral orthoses.

It has been estimated that approximately 1.5% of the world’s population (in excess of 100 million people) are in need of a prosthesis or orthosis [4]. However, it is projected at present that only 5–15% (approximately 1 in 10 persons) of the population in need has access to prosthetic and orthotic devices [5]. This short fall in access will be magnified in the future as the projected population growth and aging population will result in an increase in demand for prosthetics and orthotics [6]. Therefore, there is a need for action to accommodate the current and future demands for prostheses and orthoses.

The International Society for Prosthetics and Orthotics (ISPO) in partnership with the WHO, and funded by the United States Agency for International Development (USAID), recently published an information product in the form of Standards for Prosthetics and Orthotics Service Provision [7, 8]. These Standards were developed to encourage better access to prosthetics and orthotics services. This systematic review is part of the overall work commissioned by the ISPO, in partnership with WHO and USAID, conducted to inform these Standards.

The priority topics, provided by the funders, for the overall work, were: 1) What is the effectiveness of prosthetics and orthotics services?; and 2) What is the cost-effectiveness of prosthetics and orthotics services?; and the participants, interventions, comparisons, and outcomes (PICO) are provided in Tables 1 and 2. For the purpose of this systematic review we considered ‘prosthetics and orthotics service’ as the provision of a prosthetic or orthotic intervention to an individual by a health care professional.

**Table 1:** PICO for priority Topic 1 effectiveness of prosthetics and orthotics services.

**PICO 1.1: Population**	People with physical impairments or limb loss or functional limitations or deformities in limb or spine
**Intervention**	Provision of prosthetics/orthotics services
**Comparator**	Non-provision of prosthetics/orthotics services or provision of alternative assistive products (such as crutches, walkers, sitting board with castors, wheelchairs, and tricycles).
**Outcomes (primary)**	Coverage or access to services; prevention of fall/injuries; prevention of deformities or secondary health conditions; avoidance of premature deaths; disability adjusted life years (DALY)/ quality-adjusted life years (QALY); better health outcomes (functioning and quality of life); mobility and safety; user’s satisfaction; cosmesis/image; building human capacity; time and physical burden for care services or givers.
**Outcomes (secondary)**	Human rights; empowerment; economical gain for individual and family; independence; self-confidence and self-esteem; educational and

**Table 2:** PICO for priority Topic 2 cost-effectiveness of prosthetics and orthotics services.

**PICO 2.1: Population**	People with physical impairments or limb loss or functional limitations or deformities in limb or spine
**Intervention**	Investing in prosthetics/orthotics services
**Comparator**	Non-investing in provision of prosthetics/orthotics services or providing alternative assistive products (such as crutches, walkers, wheelchairs, tricycles) or providing social support services (such as pension, social security, provision of caregiver/assistant).
**Outcomes (primary)**	Cost and cost-effectiveness; coverage or access to services; duration of stay in hospital/clinics; clinical output; prevention of secondary deformities or health conditions; completing the rehabilitation cycle or continuum of care; cost of future treatment of falls/injuries/secondary health conditions/deformities; better health outcomes (functioning and quality of life); user’s satisfaction and availability of quality prosthesis/orthosis at an affordable cost; building human capacity; time and physical burden for care services or givers.
**Outcomes (secondary)**	Economical gain for the establishment/state; economical gain of individual and family; upholding human rights, educational and job opportunities; empowerment; social acceptance; participation and inclusion.

The overall work incorporated an extensive search to identify all relevant subject matter related to Prosthetics and Orthotics, and allow us to catalogue all the medical conditions in which research on prosthetic and orthotic interventions has been completed. The objective of this systematic review, as a sub group analysis of the overall work, was to identify and review the evidence from randomised controlled trials (RCTs) to assess effectiveness and cost-effectiveness of prosthetic and orthotic interventions. While an extensive number of systematic reviews have examined the specific prosthetic [9–15] and orthotic [16–20] interventions in the treatment of specific medical conditions and/or injuries, as such no one has completed an overall examination of orthotic and prosthetic interventions across healthcare. This review also provides an opportunity to provide an update to previous systematic reviews in the area, as more recent research is now available for many of the previously published systematic reviews.

## Methods

The systematic review was performed according to the PRISMA guidelines (see S1 Table) [21]. It was registered in the PROSPERO database for systematic reviews (PROSPERO, registration number CRD42016025994) [22], this protocol is provided in S1 File.

### Search strategy

MeSH headings and free text terms for orthotics and prosthetics along with study design categories were used to capture all research in this area; with 14 databases searched: Web of Science, Medline, PubMed, CINAHL Plus, EMBASE, SCOPUS, Rehabdata, PsycInfo, ERIC, Education Research Complete, Business Source Complete, IEEE, NIHR and CEA Registry. A sample search terms and strategy for MEDLINE is provided in S2 File, and searches were adapted for each database. No language restriction was applied to the search and the searches were completed between 22^nd^ and 27^th^ September 2015. While resources were not available to enable the inclusion of non-English language articles in this manuscript no language restrictions were applied to the search to enable a complete database of research in the area to be established.

### Study selection and data extraction

The search results were independently screened by two reviewers. Inclusion criteria were as follows: studies which (1) provided devices (orthotic/prosthetic) for a clinical problem and for use during activities of daily living, (2) involved participants of all age and any medical condition, and (3) used valid outcome measures (defined as measures which are described in detail with references provided where applicable, and which have been validated for use on the clinical population being examined). Exclusion criteria were as follows: (1) Case series/report/studies; expert opinion articles; letters to the editor; commentaries; cross-sectional studies, and studies (2) involving healthy participants, (3) examining devices for prevention of injuries and within therapy/training sessions and (4) examining novel/research devices. While the authors acknowledge that a large volume of cross-sectional studies have been published within this field, as identified within our literature search, it is widely accepted that the cross-sectional study design is considered to offer low levels of evidence. Hence, for this manuscript the authors decided to focus on RCTs and exclude these studies.

Online screening software (Covidence systematic review software, Veritas Health Innovation, Melbourne, Australia. Available at www.covidence.org) was used to complete the independent screening, with discrepancies between reviewers resolved through discussion and advice from a third reviewer where needed. Data extraction from the included studies was completed by one of three reviewers, with a second reviewer checking the extracted data for accuracy and completeness. Any identified discrepancies were then discussed by the reviewers to ensure the accuracy of the extracted data. The data extracted from the included studies were: (1) the clinical population examined, (2) the type of orthotic/prosthetic intervention, (3) the comparator/s and (4) the outcome measures. Studies were grouped based on their clinical population (medical condition/injury) and each clinical population was sub categorised based on the comparator interventions. For each clinical population, there were three sub categories:

- Category A: Provision of a prosthetic/orthotic Vs. non-provision
- Category B: Provision of a prosthetic/orthotic Vs. prosthetic/orthotic comparator
- Category C: Provision of a prosthetic/orthotic Vs. non-prosthetic/orthotic comparator

#### Risk of bias

The included articles were assessed for risk of bias using the Cochrane Risk of Bias tool [23]. Two review authors independently assessed risk of bias of the included studies with disagreements between reviewers resolved through discussion and advice from a third reviewer where needed.

### Data synthesis

Data synthesis was completed as previously reported by Farmer et al. [24]; as the heterogeneity in the participants and treatment interventions were considered too great to combine data in a meta-analysis, each RCT was treated as an individual data point. The extracted data were used to calculate effect sizes (ES) or odds ratios (OR), where appropriate, for all outcome measures of each study separately. Due to the nature of this review which was to inform the development of standards for orthotic and prosthetic services, it was imperative to extract all valid outcome measures from the identified RCTs.

The equations used to calculate effect size (Equation 1), the standard error of the effect size (Equation 2), and odds ratio (Equations 3–5) are provided in S3 File. For the calculated effect sizes, if the range for the 95% Confidence Interval (CI) did not include zero, then the result was considered statistically significant. The sample sizes used for the equations were the number of participant who completed the study, unless it was stated by the authors that they used intention to treat when completing their statistical analysis. The calculated effect sizes and odds ratios for the outcome measures of each of the included studies are provided in S4 File and S5 File, respectively.

The individuals in the figures in this manuscript has given written informed consent (as outlined in PLOS consent form) to publish these case details.

## Results and discussion

**Fig 1:**
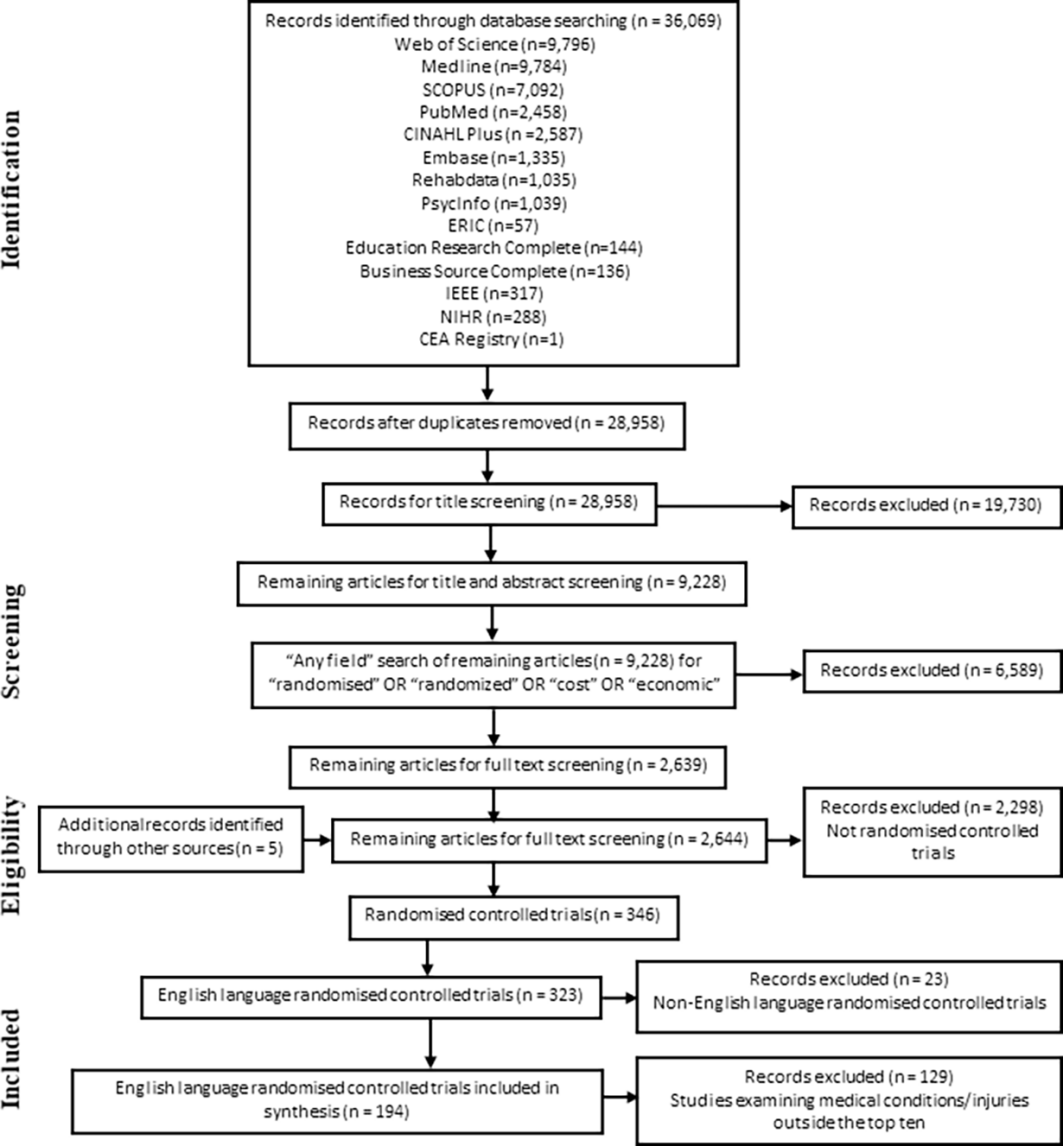
Flow diagram for selection of studies included in the systematic review.

A flow chart outlining the study selection is presented in Fig 1. The database searches resulted in a total of 28,958, initial screening by two reviewers reduced the total to 9,228. While the objective of the overall work was to identify all research in the area of prosthetic and orthotic services, the objective of this manuscript was to focus on locating the studies with the highest level of evidence (RCTs) and those which examined cost-effectiveness. The decision was based on the rationale that potential bias is likely to be greater for non-randomised studies and their results should be interpreted with caution. Therefore, the conclusions presented within this manuscript would be stronger if limited to RCTs.

To locate these studies an “Any field” search within Endnote reference manager (Version X7. Available at www.endnote.com) of the remaining 9,228 results for the words “randomised” OR “randomized” OR “cost” OR “economic” was conducted, and this reduced the total to 2,639. The titles and abstracts of these 2,639 studies were then independently screened by two reviewers. Within these studies were published protocols from which an additional 5 potential studies were identified for screening, giving a total of 2,644 studies. From these 2,644 studies selected for review, 346 randomised controlled trials were identified, of which 323 were English language. Of the 323 English language RCTs, 319 examined orthotic interventions and 4 examined prosthetic interventions.

The results, presented below, have been categorised with separate sections providing the results and discussion for the included prosthetic and orthotic intervention studies. The section on orthotic interventions has been further sub-sectioned based on the clinical population of the included studies. The final section provides a general discussion on all the included studies and identifies issues which require consideration if future research is to enhance the quality of research in this area.

### Prosthetic interventions

**Fig 2:**
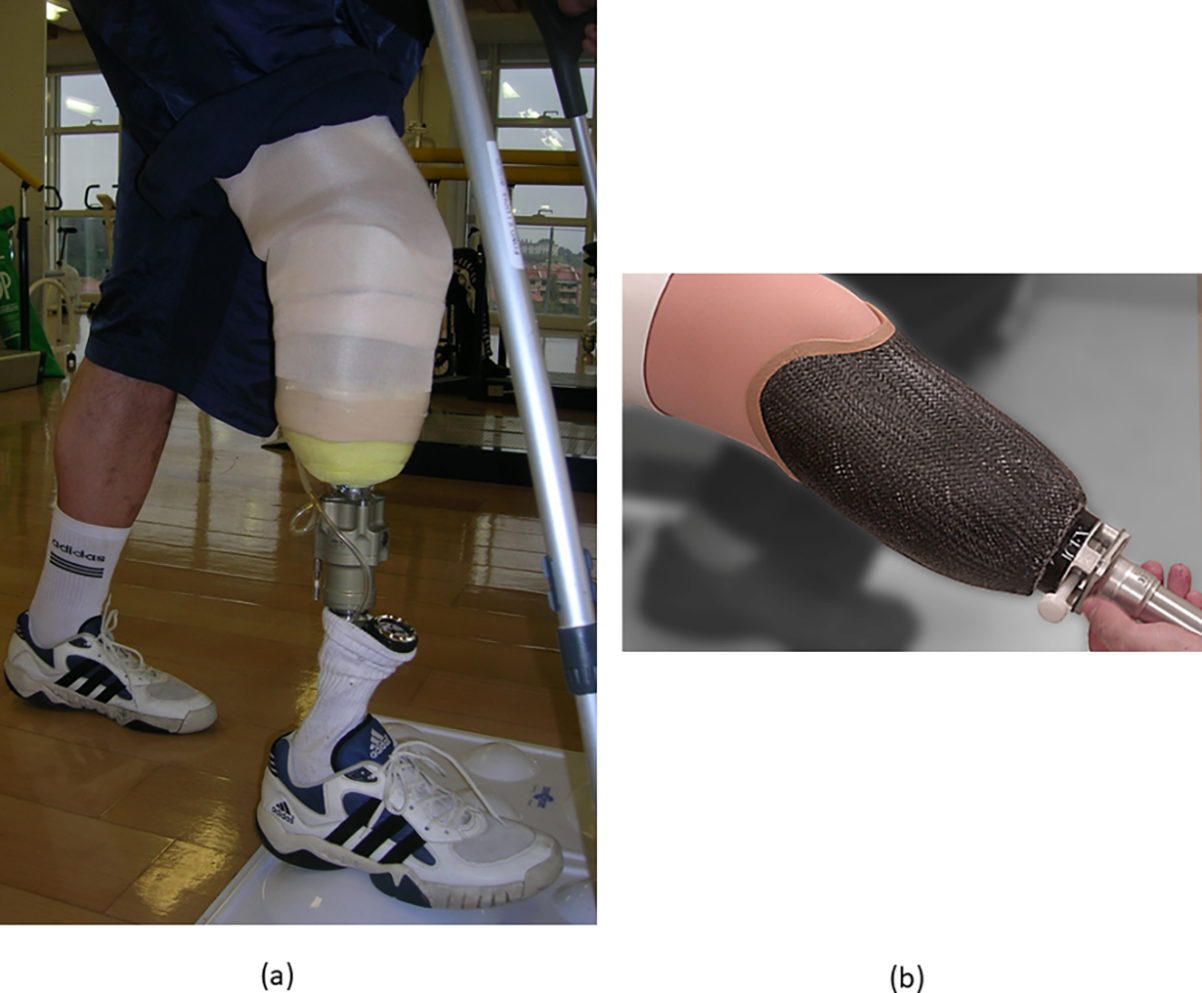
Examples, from the studies included in the systematic review, of prosthetic interventions. Prosthesis training with Vacuum Assisted Socket System (Harmony^®^). In the early stages of adapting, the pressure gauge is maintained to control the vacuum. At a later stage it will be removed and the prosthesis refined (a) [25] (image courtesy of Dr Stefano Brunelli); ICEX socket (image courtesy of Össur, UK) (b).

Four RCTs were identified which examined prosthetic interventions [25–28], with all four studies examining lower limb prosthesis. These four studies examined different socket systems for transtibial prostheses with two studies providing data which allowed for effect size calculations [26, 27], see S4 File for further details. These two studies compared the ICEX total surface bearing socket to a patellar tendon bearing socket (Fig 2). Datta et al. [27] examined a range of gait parameters with non-significant small and medium effect sizes calculated for all the outcome measures. Selles et al. [26] also compared a range of gait parameters with non-significant small and medium effect sizes calculated for all the outcome measures.

None of the identified RCTs examined the cost-effectiveness of the prosthetic interventions. Some information on the cost of the prosthetic interventions was provided in the two studies for which data was extracted above [26, 27]. Datta et al.[27] compared the cost and Selles et al. [26] compared the cost efficiency between the two interventions; the ICEX total surface bearing socket and the patellar tendon bearing socket. Both studies concluded that while the manufacturing time for an ICEX socket was less than the patellar tendon bearing socket, the cost of the ICEX socket was greater.

#### Risk of bias

Results of the risk of bias assessment for the two studies for which data was extracted are provided in Figure A in S6 File. Neither study provided information on the randomisation or allocation concealment procedures within their studies and were therefore identified as having an unclear risk of bias. A high risk of bias for both studies was assessed for blinding, as neither the participants nor the outcome assessors were blinded. For selective reporting, it was judged that there was an unclear risk of bias for Datta [27], as some data for the control participants was not available. A low risk of bias was assessed in both studies for other sources of bias.

This review has highlighted the lack of high quality RCTs examining the effectiveness and cost-effectiveness of prosthetic interventions. The two studies from which data was extracted were rated as having a high risk of bias in many of the entries on the Cochrane risk of bias tool. Previous research which systematically reviewed microprocessor-controlled prosthetic knees also highlighted the need for additional research on prostheses [13] and their secondary analysis identified some of the methodological issues of research to date in this area [29].

### Orthotic interventions

The RCTs which examined orthotic interventions were categorised by the medical condition/injury of the sample population, with 68 categorises (Table 3) identified. For many of these categories only one RCT was identified which examined a specific medical condition/injury; for this reason, it was decided that this manuscript focused on the ten medical conditions/injuries with the highest number of RCTs (Table 4). When the medical conditions/injuries were arranged in order by those with the highest number of RCTs, to identify the top ten, at the 10^th^ position there were three medical conditions/injuries with a total of eight randomised controlled trials which examined orthotic interventions, therefore twelve medical conditions/injuries were included in the analysis. These twelve medical conditions/injuries comprise 60% of the total number of identified orthotic RCTs (194 of 319 studies). These RCTs were categorised into the three sub categories based on the comparator interventions (Categories A, B and C detailed above) and those which provided data that allowed for effect size or odds ratio calculation were identified (Table 4). A small number of studies had a study design which meant they fit into more than one of these three categories.

**Table 3:** Medical condition/injury categorises. List of the 68 categories for the RCTs which examined orthotic interventions.

Achilles Tendinopathy (7)	Finger joint hyperextension (3)	Pain (pelvis) (1)
Achilles Tendon Rupture (7)	Flatfoot (3)	Pain (shoulder) (1)
ACL (16)	Fracture (26)	Patellofemoral joint syndrome (4)
Acquired brain injury (2)	Functional Ankle Instability (2)	Perthes disease (1)
Anterior cervical fusion (2)	Hallux valgus (4)	Pes Cavas (2)
Axillary burn (1)	Knee replacement (3)	Plantar fasciitis (18)
Callosities (1)	Lateral epicondylitis (8)	Positional skull deformation (2)
Carpal tunnel syndrome (20)	Ligament rupture (4)	Pronated foot (2)
Cerebral palsy (8)	Leprosy (1)	Rheumatoid and juvenile idiopathic arthritis (13)
Cervical radiculopathy (3)	Lower back pain (8)	Scoliosis (4)
Contracture (1)	Lumbar spinal arthrodesis (1)	Sensory loss (1)
de Quervain's tendinopathy (3)	Mallet finger (5)	Sever's injury (2)
Developmental Coordination Disorder (1)	Medial tibial stress syndrome (1)	Shin splints (1)
Developmental dysplasia (1)	Meniscus repair (1)	Spinal cord injury (1)
Diabetes (15)	Metatarsalgia (3)	Spinal paraplegia (1)
Dislocation—knee (2)	Metatarsus adductus (1)	Sprain (10)
Dislocation—shoulder (4)	Musculoskeletal problems (1)	Stroke (22)
Digital nerve injury (1)	Neurological condition (1)	Symphysis pubis dysfunction (1)
Downs syndrome (1)	Osteoarthritis (30)	Talipes (Clubfoot) (1)
Duchenne muscular dystrophy (1)	Osteoporosis (3)	Thumb surgery (2)
Dupuytren Contracture (3)	Overuse injury (3)	Trigger finger (1)
Elbow stiffness (1)	Pain (foot) (4)	Whiplash (6)
Extensor tendon injury (3)	Pain (knee) (2)	

**Table 4:** Studies within each medical condition/injury. Breakdown of number of studies within each medical condition/injury, and the number of studies within each category for which data was extracted.

	Medical condition / Injury	Total number of RCTs	Number of RCTs from which data was extracted / Number of RCTs in Category A	Number of RCTs from which data was extracted / Number of RCTs in Category B	Number of RCTs from which data was extracted / Number of RCTs in Category C
1	Osteoarthritis	30	5/9	15/20	1/1
2	Fracture*	26	5/9	2/3	4/15
3	Stroke*	22	8/10	7/7	3/7
4	Carpal tunnel syndrome	20	3/5	3/5	3/10
5	Plantar fasciitis*	18	1/4	9/11	3/4
6	Anterior cruciate ligament (post-surgery)	16	7/12	2/3	0/1
7	Diabetic foot*	15	4/4	6/7	5/6
8	Rheumatoid and juvenile idiopathic arthritis	13	6/8	5/5	0/0
9	Ankle sprain*	10	0/0	3/5	2/5
10	Cerebral Palsy	8	1/5	2/2	1/1
	Lateral Epicondylitis	8	0/2	0/1	4/5
	Low back pain*	8	4/5	3/3	0/1
	**Total**	**194**	44/73	57/72	26/56

RCTs: randomised controlled trials; Category A: Provision of a prosthetic/orthotic Vs. non-provision; Category B: Provision of a prosthetic/orthotic Vs. prosthetic/orthotic comparator; Category C: Provision of a prosthetic/orthotic Vs. non-prosthetic/orthotic comparator.

*Some studies for this medical condition/injury were categorised to more than one category.

Study characteristics of the RCTs for which data was extracted along with the calculated effect sizes and 95% confidence intervals are available in S4 File, with odds ratios and 95% confidence intervals available in S5 File.

#### Risk of bias

Details on the assessment of the risk of bias of all studies for which data was extracted are available in S6 File; with an overall figure for all orthotic studies (Figure B in S6 File) and individual figures for each of the medical conditions/injuries (Figures C-N in S6 File). Regarding randomisation there were wide discrepancies in reporting among the medical conditions/injuries. Only the RCTs examining orthotic interventions for ankle sprain, lateral epicondylitis and low back pain provided appropriate descriptions of the randomisation procedures for all studies. There were several RCTs which used quasi-randomisation or did not state the method of randomisation. Similarly, just over 60% of the RCTs provided adequate allocation concealment with nearly 30% of studies not stating any information on concealment. For most of the RCTs it was not possible to blind the participant to the intervention provided to them, given the type of interventions provided within this area of research. Blinding of participants was only possible for some of the medical conditions which provided foot orthosis (arthritis, plantar fasciitis, diabetic foot, cerebral palsy and low back pain). For most of the RCTs the outcome measures were self-reported, therefore outcome assessors were not blinded and there was a high risk of bias for detection bias. Most studies reported missing data with balanced numbers across interventions groups, however, many studies did not employ an intention-to-treat analysis. Regarding selective reporting, limited outcome data or insufficient information to make a judgement was evident in several studies. Across all medical conditions, except lateral epicondylitis, there was a high number of studies deemed to have an unclear or high risk of bias from other sources. This reasons for this included contradicting information within the conclusion section, insufficient detail provided on the intervention(s)/outcome measure(s), insufficient detail on statistical analysis procedures, and demographic differences between intervention groups.

#### Arthritis

Forty-three RCTs were identified which examined the use of orthoses in the treatment of individuals with arthritis [30–72]. Thirty of these studies examined individuals with osteoarthritis and thirteen examined individuals with rheumatoid and juvenile idiopathic arthritis. Three of these thirteen rheumatoid and juvenile idiopathic arthritis studies examined a paediatric population [38, 46, 61], with the remaining studies examining an adult population. Data extraction for effect size calculation was possible in thirty-two of the forty-three studies. These studies had sample sizes ranging from 30 to 200 participants and follow up periods between 4 weeks and 2 years. An extensive range of outcome measures were reported in these studies as they examined a wide range of orthotic interventions for different regions of the body.

**Fig 3:**
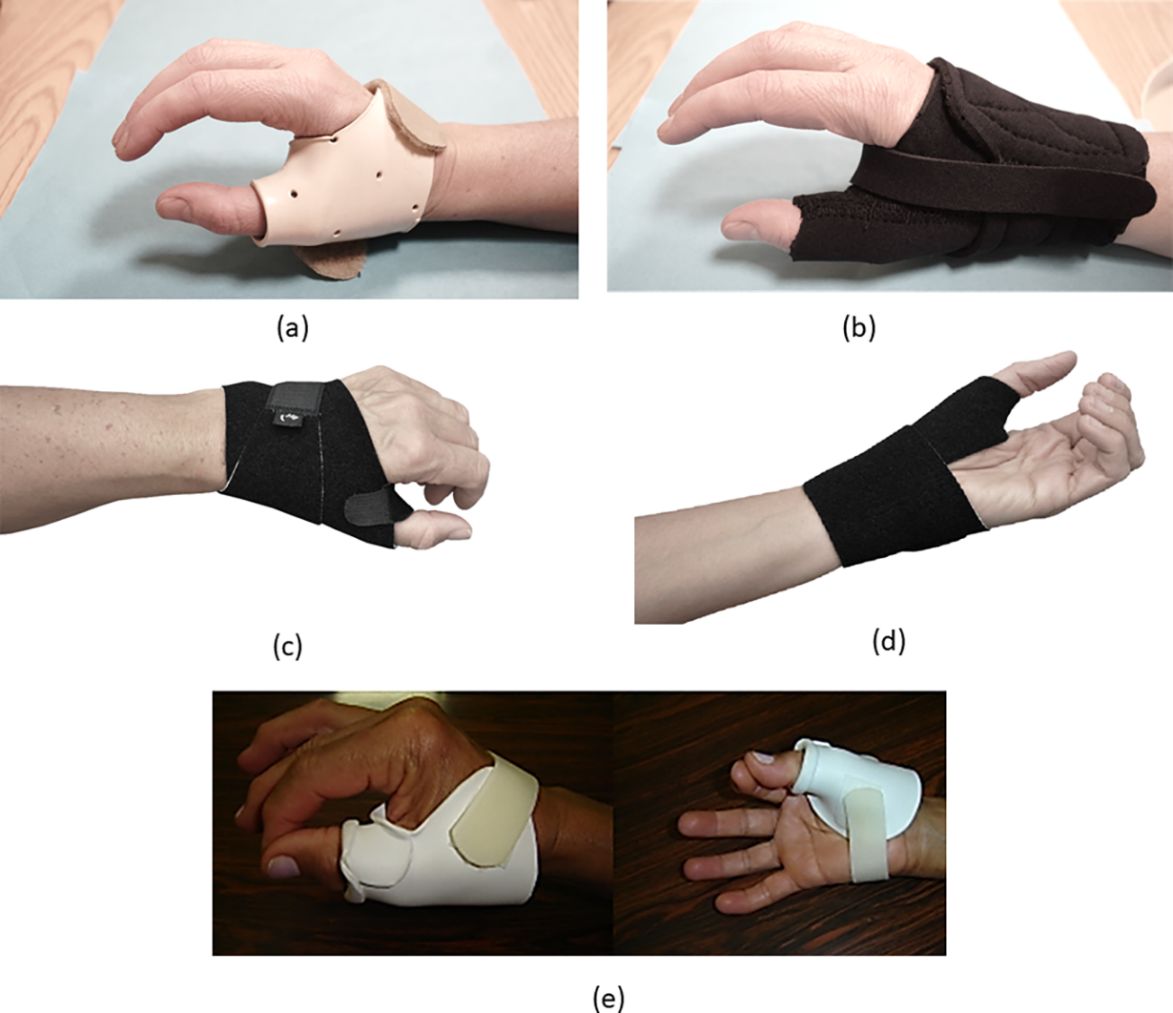
Examples, from the studies included in the systematic review, of upper limb orthotic interventions for individuals with osteoarthritis. Neoprene (a) and thermoplast (b) thumb splints [32] (images courtesy of Dr Stéphanie J.E. Becker); Fabrifoam thumb support 202 orthosis (c and d) [62] (images courtesy of Handaid AB); thermoplastic splint (e) [35] (image courtesy of Professor Anamaria Jones).

**Fig 4:**
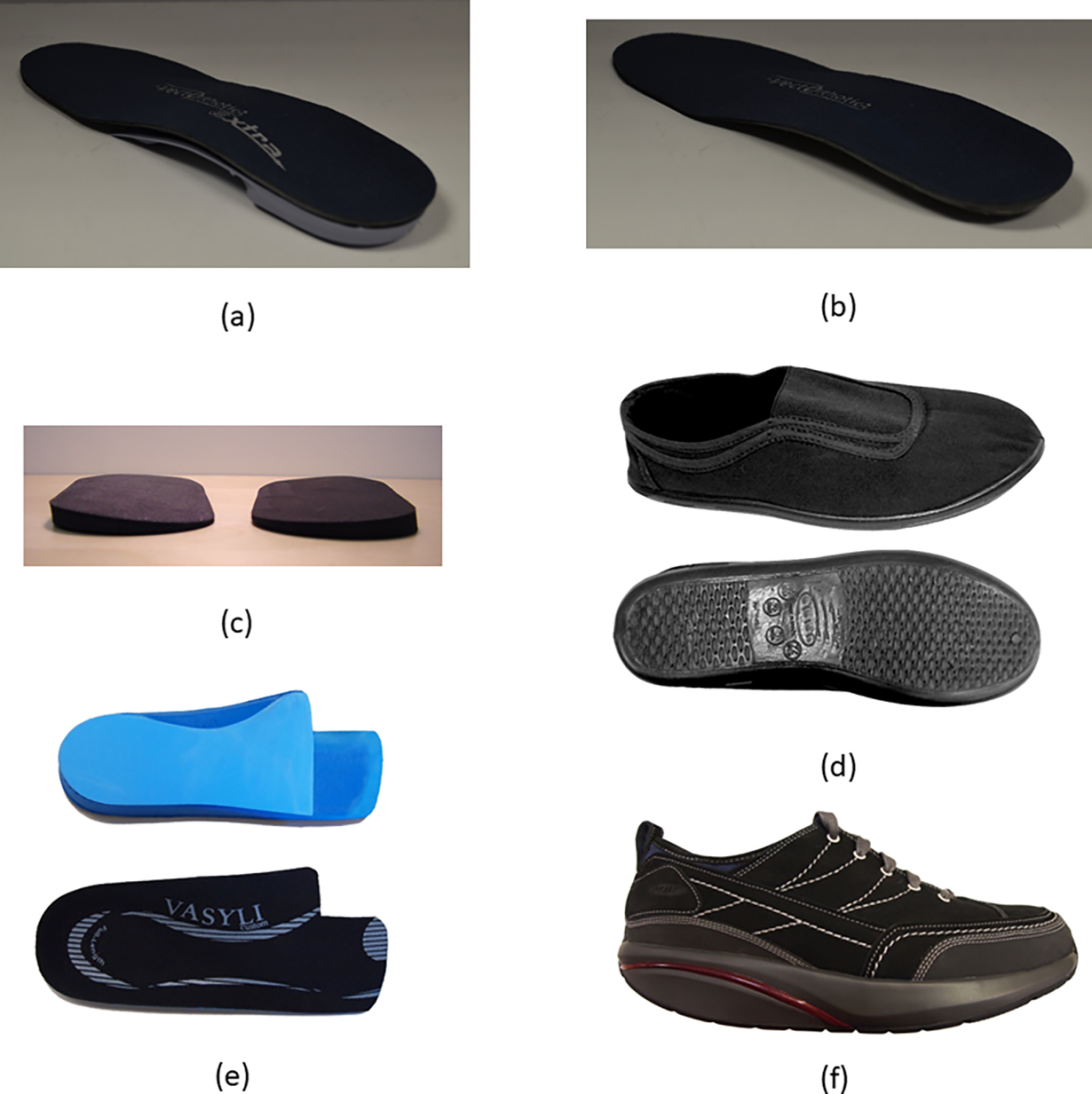
Examples, from the studies included in the systematic review, of lower limb orthotic interventions for individuals with osteoarthritis. Functional (a) and sham (b) foot orthoses [40] (images from: https://link.springer.com/article/10.1007/s10067-015-2946-6, by Halstead et al. licenced under CC BY 4.0); lateral wedge foot orthosis (c) [33] (image courtesy of Professor Kim Bennell); Moleca^®^ shoes (d) [69] (image courtesy of Professor Francis Trombini-Souza); foot orthoses (e) and rocker-sole footwear (f) [43] (images from: https://bmcmusculoskeletdisord.biomedcentral.com/articles/10.1186/1471-2474-15-86, by Menz et al. licenced under CC BY 4.0).

Data was extracted from twenty-one of the thirty studies (S4 File) which examined individuals with osteoarthritis (5 in Category A, 15 in Category B and 1 in Category C). Most studies examined orthotic interventions for the foot (15 studies), with the remaining studies examining the hand (4 studies) and the knee (2 studies) (Figs 3 and 4). In Category A, Trombini-Souza et al. [69] compared a group provided with low-cost minimalist footwear to a control group. No differences were evident between the groups for the 6-minute walk test or the Lequesne score. Skou et al. [68] compared the provision of insoles as part of a treatment program which also included neuromuscular exercise, patient education, dietary advice and prescription of pain medication to a control group which were provided with two leaflets with information and advice on osteoarthritis and recommended treatments. No differences were evident between the group for the primary outcome measure (KOOS_4_, the average score for the subscale scores of the Knee injury and Osteoarthritis Outcome Score). Also, no differences were evident for any of the subscales of the KOOS (activities of daily living, pain, quality of life, sport/recreation and symptoms). A significant small effect size (ES: 0.43 (95% CI 0.04 to 0.82) for the EuroQol health questionnaire results indicated a superior improvement in health status in the control group. No differences were evident for the remaining secondary outcome measures (20m walk test, timed up and go, EQ VAS, and bodyweight change).

Category B contained twelve studies which compared different foot orthotic interventions. No differences were found, as evidenced by non-significant small effect sizes, for four studies which compared a flat to a wedged foot orthosis [33, 40, 42, 45]. Two of these studies [42, 45] examined the same participants at two different follow-up periods (6 and 24 months). Similarly, no differences were found in Menz et al. [43] who compared a wedged foot orthosis to rocker sole footwear and Rafiaee and Karimi [48] who compared two types of wedged foot orthoses (3mm vs. 7mm). Rodrigues et al. [49] compared a flat to a medially wedged foot orthosis, with no differences evident for the majority of the outcome measure (Lequesne index, pain, pain (at night) and Western Ontario and McMaster Universities Arthritis Index). However, a significant very large effect size (ES: -1.34 (95% CI -2.46 to -0.22)) indicated that the wedged orthosis was superior to the flat orthosis in reducing pain on movement. van Raaij et al. [55] compared a knee brace to a laterally-wedged foot orthosis with no differences evident between the interventions for pain reduction, WOMAC (function) or Hip-Knee-Ankle angle.

Four studies from the same research group [51–54] examined a range of foot orthosis interventions with some designed specifically for Japanese participants as they stated that the majority of Japanese people wear shoes outdoors, but not indoors. These four studies examined the use of an orthosis strapped to the foot for indoor use, different orthosis wedge heights, different orthosis wear times and the use of an orthosis within different footwear. For all four studies no differences were evident across the tested interventions for any of the reported outcome measures (femorotibial angle, Lequesne index and pain). The single study in Category C compared acupuncture to laterally wedged foot orthosis [30] with no superiority of one intervention over the other for the reported outcome measures (pain, WOMAC, femoral cartilage thickness and tibial cartilage thickness).

Of the four studies, which examined hand orthoses in individuals with osteoarthritis two compared thumb orthoses to a control condition (Category A) [62, 66] while two compared two thumb splint interventions (Category B) [32, 35]. Within Category A, no differences were evident between the control and intervention group in Hermann et al. [62], who compared grip and pinch strength and pain outcome measures. In Rannou et al. [66] superior results, as evidenced by a significant small effect size, was calculated for the orthosis group for the Kapandji index (thumb opposition score) but no difference was evident for the Kapandji index (thumb counter-opposition score). No differences were evident for the remaining outcome measures (patient global perceived disability, Cochin hand functional scale, closure of the first web, Kallman score, pinch strength and pain). In Category B, Becker et al. [32] compared two thumb splints (neoprene vs. thermoplast) with no differences found, as evidenced by non-significant small effect sizes, for all outcome measures (Disabilities of the Arm, Shoulder, and Hand instrument (DASH), pain and grip and pinch strength). Carreira et al. [35] compared a group provided with a thumb splint for 90 days to a group who only used the splint for evaluation. While the group who used the splint for 90 days showed superior results in terms of pain reduction (significant large ES: -1.1 (95% CI -1.90 to -0.30)), non-significant small effect sizes were calculated for the remaining function and dexterity measures (grip and pinch strength, upper limb dexterity and DASH).

The first of two studies which examined knee orthoses in individuals with osteoarthritis compared a knee brace group to a control group [58]. The knee brace group had superior pain reduction (significant medium ES: -0.75 (95% CI -1.16 to -0.34)) but no differences in KOOS and patellofemoral bone marrow lesion volume results. Chen et al. [36] compared a magnetic knee brace to a placebo knee brace with no differences evident between the interventions for the outcome measures (isokinetic flexion torque and health assessment questionnaire).

Very few significant differences were evident between the intervention groups in the included studies across all the orthotic interventions for osteoarthritis. Pain was the outcome measure were most of the significant differences were evident. One Cochrane review has been completed in this area, it examined the use of braces and orthoses for the treatment of osteoarthritis of the knee [73]. In line with the findings from the current review, the authors reported inconclusive evidence for the benefits of orthotic interventions in treating knee osteoarthritis.

**Fig 5:**
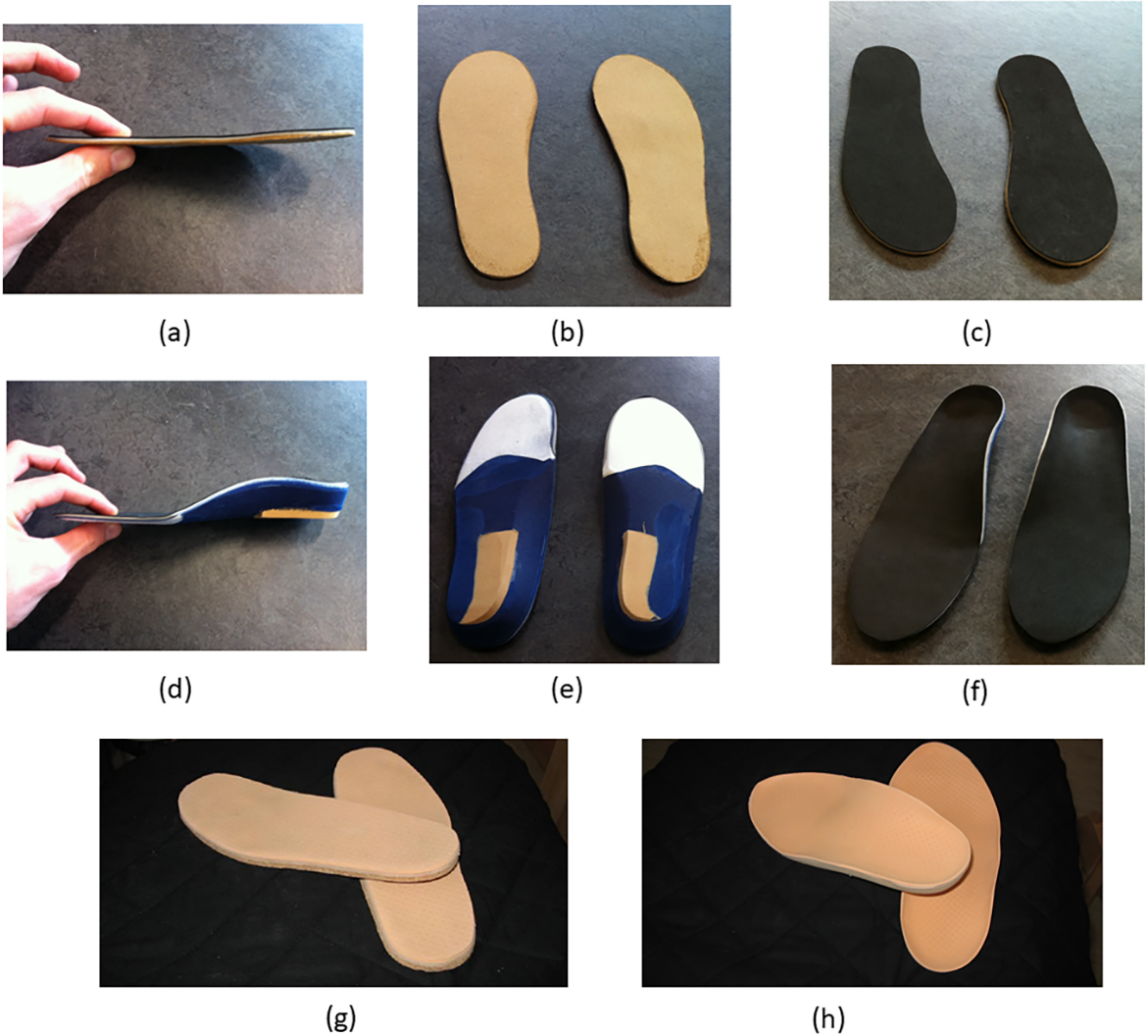
Examples, from the studies included in the systematic review, of orthotic interventions for individuals with rheumatoid arthritis and juvenile idiopathic arthritis. Control (a, b and c) and custom (d, e and f) foot orthoses [38] (images courtesy of Dr Andrea Coda; Control (g) and custom (h) foot orthoses [44] (images courtesy of Dr Primoz Novak).

Data was extracted from eleven of the thirteen studies (S4 File) which examined individuals with rheumatoid and juvenile idiopathic arthritis (6 in Category A and 5 in Category B). Seven of these studies examined orthotic interventions for the foot and two studies each examined the hand and the wrist (Fig 5). Three studies compared orthotic interventions for the foot (foot orthosis or footwear) to a control condition [60, 61, 71], with no differences identified for any of the reported outcome measures (Childhood Health Assessment Questionnaire, Juvenille Arthritis Foot Disability Index, EuroQol health questionnaire, Disease Activity Score, Stanford Health Assessment Questionnaire, Foot Function Index, walking cadence and velocity, Larsen index joint erosion score, fatigue, pain and wellbeing). While these studies utilised a range of different outcome measures they all examined the effect of the interventions on pain reduction.

Both Coda et al. [38] and Novak et al. [44] compared a foot orthosis to a control foot orthosis with no differences evident between the interventions for the reported outcome measures (6 minute walk test, Foot Function Index, Paediatric Quality of Life questionnaire and pain). Similarly no differences were evident between the soft and semi-rigid custom foot orthosis groups in Cho et al. [37]. Powell et al. [46], compared three interventions (custom foot orthosis, prefabricated foot orthosis and athletic footwear), and measured a range of outcome measures. While no differences were evident between any of the three interventions for pain, Foot Function Index and the Paediatric Quality of Life questionnaire, the custom foot orthosis group were superior to the prefabricated foot orthosis group for a reduction in a timed 50-foot walking test (significant large ES: -1.03 (95% CI -1.87 to -0.19)).

The two RCTs which examined the use of wrist splints [56, 70] compared them to a control condition. No differences were evident for the outcome measures (Disabilities of the Arm, Shoulder, and Hand instrument, grip strength and Sequential Occupational Dexterity Assessment) reported by Veehof et al. [70]. Opposing findings were evident from Adams et al. [56], with a very large effect size (ES: 1.35 (95% CI 0.96 to 1.74)), indicating superior pain reduction in the control group compared to the wrist splint group. No differences were evident for the remaining outcome measures (dexterity, ulnar deviation, grip strength, stiffness and Michigan Hand Outcomes Questionnaire).

The two RCTs which examined the hand [50, 67] were completed by the same research group. One study compared the use of a thumb orthosis to a control group with superior results, evidenced by significant medium to large effect sizes, for the intervention group for some of their outcome measures (pain, pinch strength and Stanford Health Assessment Questionnaire). No differences were evident between the interventions for the Disabilities of the Arm, Shoulder, and Hand instrument or grip strength. The second study compared a group which used the orthosis daily to a group which only used the orthosis during the evaluation. No differences were evident between groups any of the outcome measures (pain, grip and pinch strength, upper limb dexterity and Health Assessment Questionnaire).

For the most part, the results from the included studies do not support the use of orthoses for the treatment of rheumatoid and juvenile idiopathic arthritis with contrasting findings from some studies. Findings from a Cochrane review published in 2003 [74], which examined orthotic interventions in rheumatoid arthritis, reported on the lack of insufficient evidence and stated that research in this area was in its infancy. While research in this area had increased significantly in the time since this Cochrane review, it is still not currently possible to establish a conclusive answer on the effectiveness of these interventions.

#### Fracture

Twenty-six RCTs examining the use of orthoses in the treatment of fractures were identified [75–100]. These studies included ten on the upper limb, with eight examining the arm and two examining the hand. Nine studies were conducted on the lower limb, with seven examining the ankle and two investigating tibial stress fractures. Seven studies were conducted on the spine, with four examining compression or burst fractures and three assessing osteoporotic fractures. Five of these twenty-six studies examined a paediatric population; with four studies examining arm fractures and one examining ankle fractures. Data was extracted from ten of these twenty-six studies (effect size calculations were completed on data from seven studies and odds ratio calculations on data from three studies), with sample sizes ranging from 23 to 137 participants and follow-up periods between 3 weeks and 2 years.

Five of the nine studies within Category A provided data for effect size calculations (S4 File). Four of these studies compared a control group to one provided with a thoracolumbosacral orthosis [93, 94, 98, 99]. No superior benefit of the tested orthoses over the control condition was evident in the two studies by Bailey et al. [93, 94] (which examined the same participants at two follow up periods; 1 year and 2 year) and the study by Shamji et al. [99] which examined participants with burst fractures. Pfeifer et al. [98] examined the use of two thoracolumbosacral orthoses in the treatment of osteoporotic spinal fractures, with superior results calculated for both orthoses over the control group for many of their secondary outcome measures but not their primary outcome measure (isometric back extensor strength). Significant large and very larges effect sizes, indicating superior results for the orthoses groups, were calculated for the angle of kyphosis, limitations of daily living (disability), limitations of daily living (self-care), loss of body height, and pain. Jongs et al. [96] examined the effectiveness of wrist splints in reducing contracture following a distal radial fracture; no differences between the control and intervention groups, as demonstrated by the non-significant small effect sizes, were evident.

Data was extracted for effect size calculations from two of the three studies within Category B (S4 File). Both these studies compared the use of two thoracolumbosacral orthoses in the treatment of osteoporotic spinal fractures. While as reported above, superior results for both orthoses over their control group were evident in Pfeifer et al. [98], no differences were evident between the orthoses. Li et al. [91] compared the Spinomed orthosis to a soft lumbar orthosis with no differences evident between the interventions.

Within Category C effect sizes were calculated for one study [84] and odds ratios for three studies [79–81]. O’Connor et al. [84] compared a plaster cast to a wrist splint for a Colles’ fracture with no differences in participant satisfaction or pain evident between the groups. A significant medium effect size was calculated for problem with cast/splint, indicating less problems in the cast group than the splint group (ES: 0.64 (95% CI 0.13–1.15)). Hansen and Hansen [79] compared participant satisfaction between three interventions for the hand; plaster cast, elastic bandage and functional brace. When compared to the functional brace group the plaster cast group were less satisfied with the intervention (OR: 0.50 (95% CI 0.04 to 5.89)), while the elastic bandage group were more satisfied (OR: 1.13 ((95% CI 0.07 to 18.99)). Harding et al. [80] compared a metacarpal brace to neighbour strapping with the brace group more likely to return to work within 3 weeks (OR: 0.04 (0.01 to 0.15)) and more satisfied with the intervention (OR: 0.80 (0.26 to 2.44)). Karimi Mobarakeh et al. [81] compared a plaster cast to a wrist splint with the splint more beneficial in terms of convenience (OR: 0.72 (95% CI 0.26 to 1.99)), but the plaster cast was more beneficial in relieving pain (OR: 1.59 (0.79 to 3.18)).

One RCT was identified which examined cost-effectiveness, von Keyserlingk et al. [88] compared the provision of a cast compared to a splint in children with wrist fractures. Results indicated that splint management was more cost-effective than casting, with the costing based on treatments cost in Canada.

From the included RCTs there is not enough evidence to indicate which methods of treatment for fractures are superior. Since 2006, several Cochrane reviews [101–105] have examined the effectiveness of orthotic interventions in the treatment or rehabilitation of different types of fractures; with all reviews concluding that there was a lack of sufficient high quality evidence to form conclusions and practice recommendations.

#### Stroke

**Fig 6:**
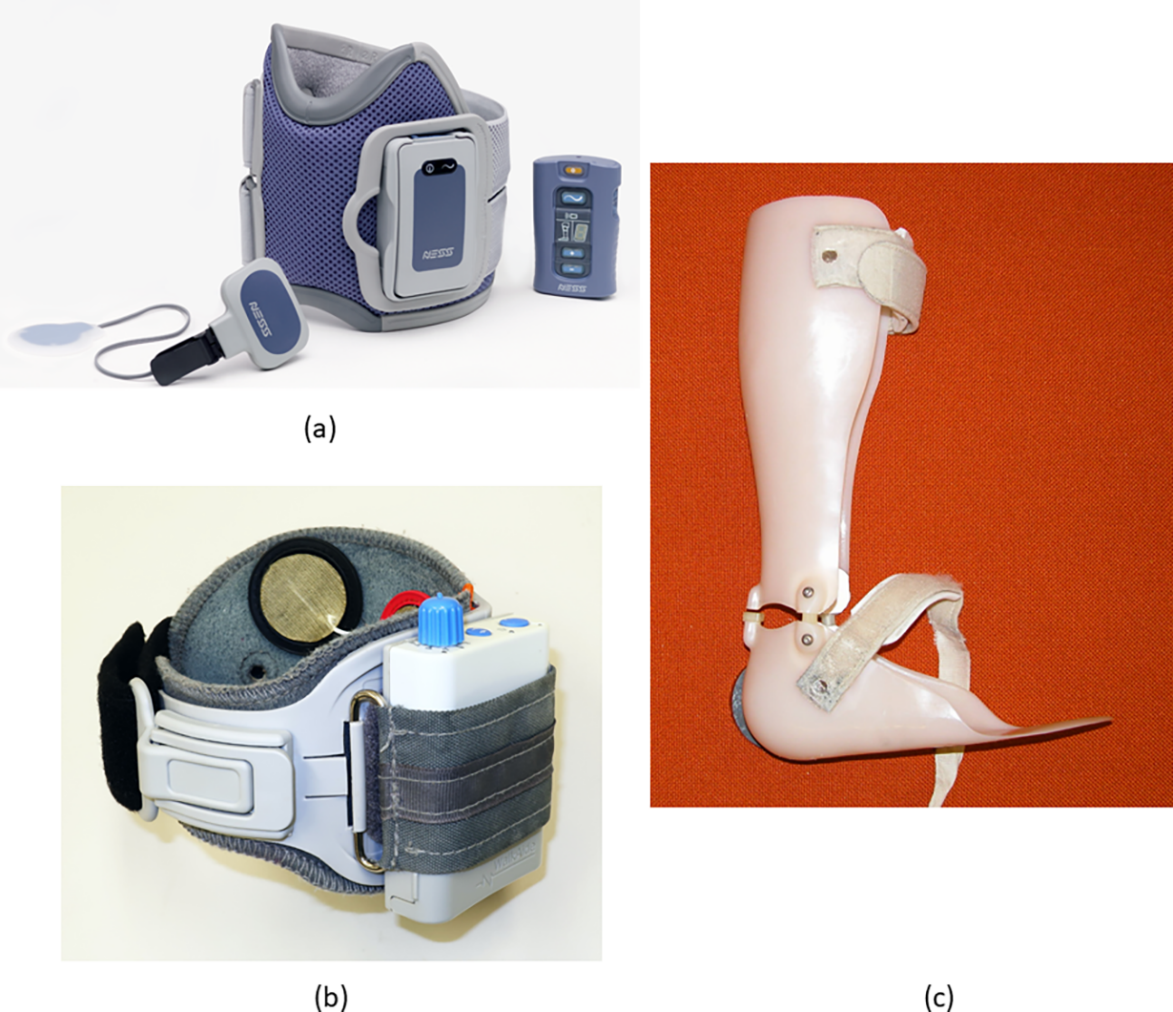
Examples, from the studies included in the systematic review, of orthotic interventions for individuals post stroke. WalkAide foot drop stimulator (a) and ankle foot orthosis (b) [115] (images courtesy of Dr Dirk Everaert); Bioness L300 foot drop stimulator [116] (image courtesy of Bioness Inc.).

Twenty-two RCTs [106–127] were identified which examined the use of orthotic interventions with individuals after Stroke. These consisted of fourteen studies examining lower limb orthotics (13 studies examining the lower leg and 1 study examining the foot), and eight studies examining upper limb orthotics (6 studies examining the wrist, and 1 study each examining the shoulder and elbow) (Fig 6). Data was extracted from eighteen of these twenty-two studies, with effect size calculations completed for seventeen studies and odds ratio calculations completed for one study. Sample sizes for these studies ranged from 9 to 197 participants and follow-up periods were between 28 days to 30 weeks.

Seven of the ten studies in Category A provided data which allowed for the calculation of effect sizes (S4 File). Three studies examined wrist orthoses and one study each examined foot, lower leg and shoulder orthoses. Few studies reported on the same outcome measures, with a wide range of outcome measures to assess gait, functional abilities and clinical symptoms utilised. Significant differences between interventions were only evident in one study; Hartwig et al. [124], who compared a control condition to a shoulder orthosis. Superior results were evident in the shoulder orthosis group for the subscales of the Shoulder-Hand Syndrome Score (significant very large ESs: ranging from -1.97 (95% CI -2.64 to -1.30) to -2.52 (95% CI -4.01 to -1.03)). Beckerman et al. [120] compared an ankle foot orthosis (AFO) to a placebo AFO, with five of the eight odds ratio calculations (for the Achilles tendon reflex, ankle clonus, muscle tone and spasticity outcome measures) indicating more participants benefited from using the AFO.

Data was extracted from all seven studies in Category B, two of which were also categorised as Category A [118, 126]. Non-significant small effect sizes were calculated for all of the reported outcome measures in the studies in this category; five studies comparing lower limb orthotic interventions (AFO, foot drop stimulator and functional electrical stimulator) and two studies comparing wrist splints.

Category C contained seven studies, with four of these reporting on the same participants [106–109]. These four studies by Kottink et al. [106–109] were excluded from data extraction as the orthotic intervention group were not provided with a specific intervention and therefore no conclusion on intervention effectiveness could be determined. The orthotic group continued using their conventional walking device during the study which consisted of a range of devices (ankle-foot orthosis, orthopaedic footwear, or no device). Data for effect size calculations was extracted from the three remaining studies. Two of these studies examined wrist splints with one making a comparison to taping [111] and the other to hybrid assistive neuromuscular dynamic stimulation (HANDS) [112]. Results from Santamato et al. [111] supported taping over the wrist splint for two of their four reported outcome measures; significant large effect size for finger position at rest (ES: -0.82 (95% CI -1.31 to -0.33)) and significant medium effect sizes for Disability Assessment Scale and Modified Ashford Scale (wrist), (ES: 0.57 (95% CI 0.10 to 1.04) and 0.75 (95% CI 0.28 to 1.22), respectively). Findings from Shindo et al. [112] did not indicate a difference between HANDS therapy and wrist splinting.

Research examining the use of orthotic interventions after stroke has examined a wide range of interventions for the lower and upper limbs. Of the included studies, only three were found to identify difference between the tested interventions; for two studies, superior results were evident for the orthotic intervention when compared to a control/placebo intervention and for one study taping was found to be superior to a wrist splint. One Cochrane review, published in 2005, examined supportive devices for preventing and treating subluxation of the shoulder after stroke [128] with the authors concluding that there was insufficient evidence on the effectiveness of the interventions. While some more recent systematic reviews have supported the use of AFOs for this clinical population [129, 130], the conclusions were based on research which only provided results on the immediate effect of the intervention. Some of the more recent systematic reviews in the area have focused on examining other treatment interventions such as functional electrical stimulation [131, 132] and Botulinum toxin A [133]. These reviews have reported positive results for the interventions, however, findings were based on limited studies often with small sample sizes and of variable methodological quality.

#### Carpal tunnel syndrome

**Fig 7:**
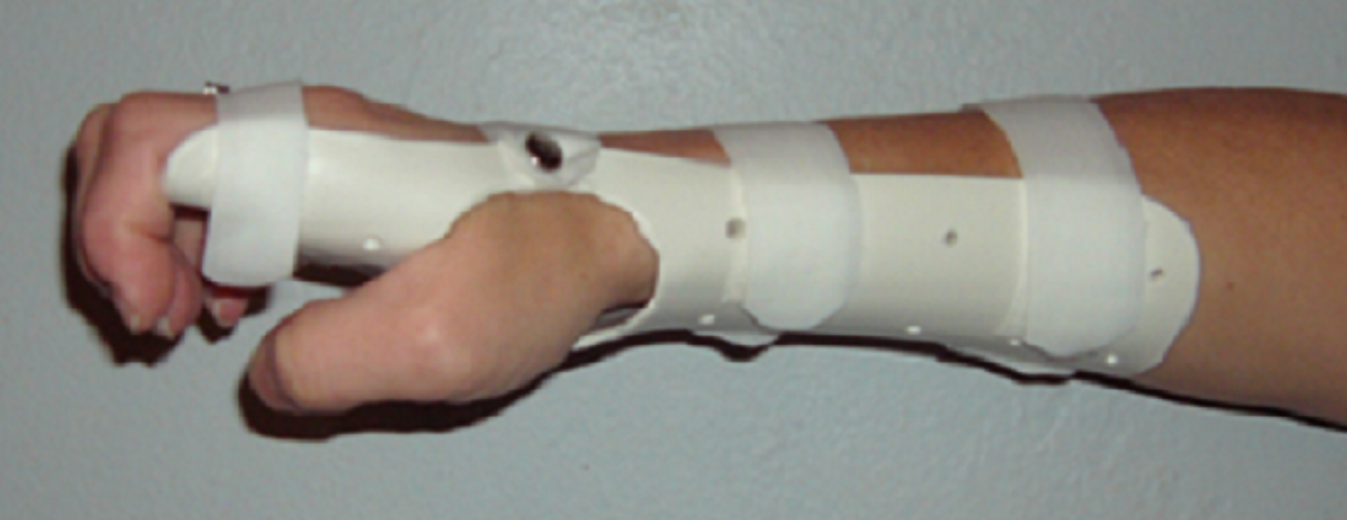
Examples, from the studies included in the systematic review, of orthotic intervention for individuals with carpal tunnel syndrome. Lumbrical splint [144] (image courtesy of Dr Nancy A. Baker).

Twenty RCTs were identified which examined carpal tunnel syndrome [134–153] (Fig 7). Seven studies were excluded from data extraction as they recruited participants with bilateral involvement [135, 137, 139, 142, 143, 148, 149], which has implications for statistical analysis [154]. Data was extracted from nine studies, with effect size calculations completed for eight studies (S4 File) and odds ratio calculations for three studies (S5 File). Sample sizes ranged from 51 to 147 participants and follow-up periods between 5 weeks and 18 months. A wide range of outcome measures were examined in these studies, with The Boston Carpal Tunnel Questionnaire Symptom Severity Scale (BQSSS) being the most utilised outcome measure.

Data was extracted from three of the five studies in Category A. While most of the results indicated superior outcomes for the groups provided with wrist splints compared to the control groups, non-significant small effect sizes were evident. For Hall et al. [150] superior results for the wrist splint group for BQSSS (significant medium ES: -0.63 (95% CI -1.18 to -0.08)) and pain (significant large ES: -0.84 (95% CI -1.53 to -0.15)) were evident. From Werner et al. [153] superior results for a reduction in discomfort (significant medium ES: -0.51 (95% CI -0.96 to -0.06)) were evident for the wrist splint group.

Within Category B, data was extracted from three of the five identified RCTs. Non-significant small effect sizes were evident for all but one of the outcomes measures, indicating minimal differences between the orthotic interventions examined. The effect sizes calculated for the outcome measures in Baker at al. [144] did not indicate large differences between the orthotic interventions, however, the odds ratio calculations, which assessed the number of participants who demonstrated a clinically important improvement in symptoms and function, indicated superior benefit for the cock-up wrist splint group over the lumbrical splint group. Results from Briniger et al. [145] supported a superior benefit for the neutral wrist and metacarpophalangeal splint over the wrist cock-up splint, for the patient reported frequency of symptoms (OR: 0.40 (95% CI 0.06 to 2.63).

Data was extracted from three out of ten studies in Category C. For the single study comparing acupuncture to night splinting [141] small and not significant effect sizes were calculated for the superior results of splinting. In contrast, results from Gerritsen et al. [138] supported surgery over splinting, with significant small effect sizes calculated for BQSSS, day paresthesia and severity of main complaint (ES: ranging from 0.39 (95% CI 0.06 to 0.72) to 0.47 (95% CI 0.18 to 0.76). The odds ratios calculated for the outcome measures reported by Bardak et al. [134] supported the use of wrist splints and local steroid injections over nerve gliding exercises, along with a significant large effect size for a superior reduction in symptom total point for the splinting group (ES: -1.12 (95% CI -1.65 to -0.59). The mean age of the participants in the intervention groups in Bardak et al. [134] were considerably younger (22 and 33 years) than the other RCTs whose participants had a mean age of between 44 and 54 years.

One RCT was identified which examined cost-effectiveness; Korthals-de Bos et al. [140] reported that surgery was more cost-effective than splinting for carpal tunnel syndrome in the Netherlands.

Results from the studies identified in this review are inconclusive, with contrasting findings across studies. A Cochrane review published in 2012 [19], examined the use of splinting in the treatment of carpal tunnel syndrome, highlighted the insufficient evidence in this area. The RCTs identified by this Cochrane review and the present review differed as there were differences in the selection criteria for the reviews; the Cochrane review included non-English language studies, searched different databases, had different inclusion/exclusion criteria and included RCTs from different publication years to the present review. The present review identified five RCTs completed since the Cochrane review was published, however, these studies have not added significantly to the evidence in this area as there was a high risk of bias in the two studies from which data as extracted and two studies were excluded as they recruited participants with bilateral involvement and included both hands in their assessment.

#### Plantar fasciitis

**Fig 8:**
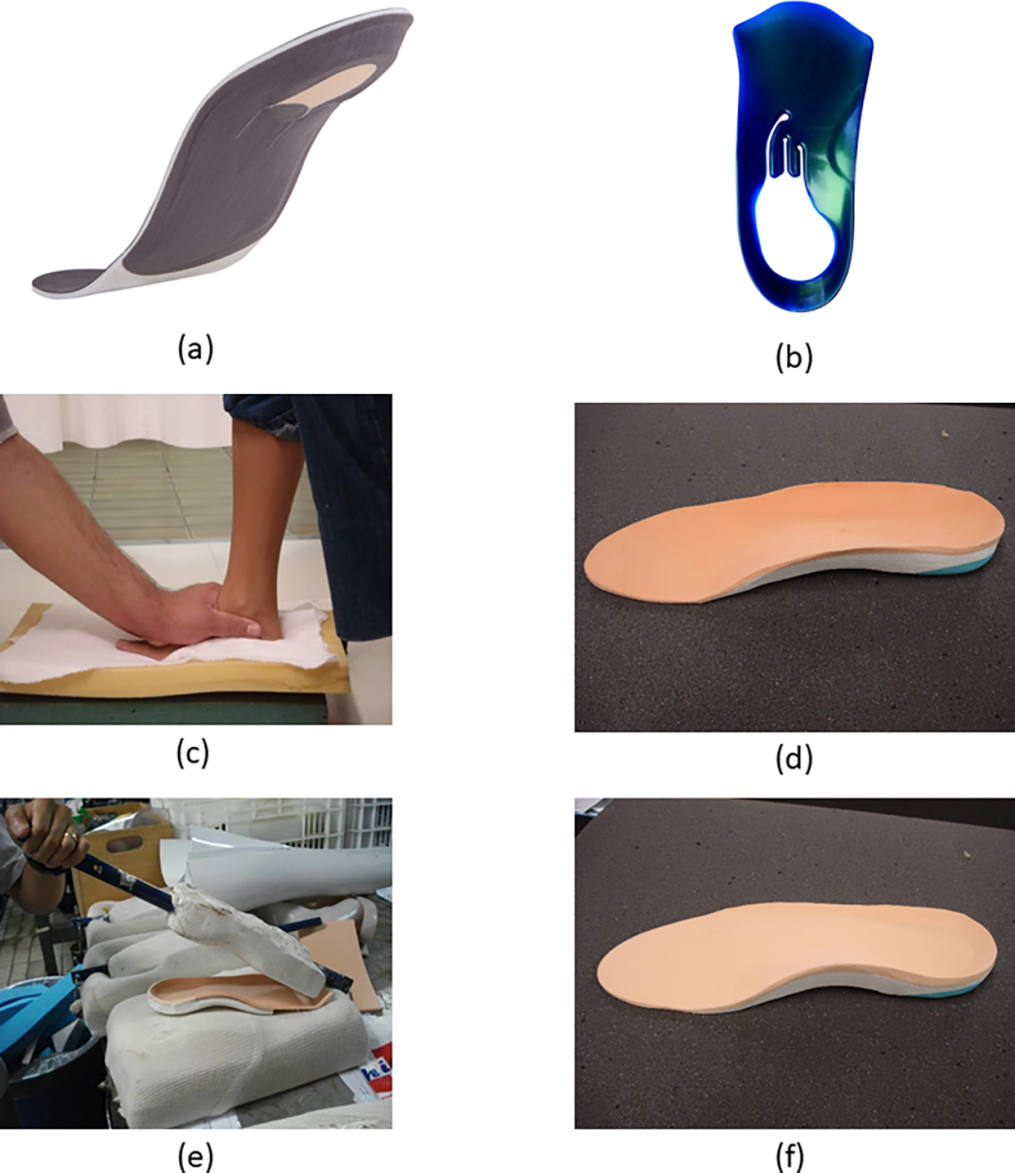
Examples, from the studies included in the systematic review, of orthotic interventions for individuals with plantar fasciitis. ErgoPad redux foot orthosis (a and b) [168] (images courtesy of Bauerfeind AG); custom (c and d) and prefabricated (e and f) foot orthoses [159] (images courtesy of Ms Valéria Baldassin).

Eighteen studies were identified which examined orthotic interventions for the treatment of plantar fasciitis [155–172] (Fig 8). Data was extracted from twelve of these eighteen studies (S4 File), with sample sizes ranging from 30 to 200 participants and follow-up periods of between 3 weeks and 12 months. Three studies were excluded from data extraction as they recruited participants with bilateral involvement [169, 171, 172], which has implications for statistical analysis [154]. One study was excluded as nearly 30% of the participants had their treatment terminated during the study [162]. All but three of the studies for which data was extracted examined a foot orthosis (prefabricated/custom foot orthosis or heel pad/cup) with the other studies examining night splints [163, 166], the combined treatment of a foot orthosis and a night splint [166] and an ankle brace [160].

One study from Category A provided data for effect size calculation, with no differences evident between the control condition and any of the three interventions (custom foot orthosis, silicone heel pad and felt pad) for the Foot Function Index (FFI) pain subscale [170]. This study was also categorised to Category B, with no differences evident between the orthotic interventions.

Nine of the eleven studies in Category B provided data which allowed for the calculation of effect sizes [159–161, 163–166, 168, 170]. Many of these studies used different outcome measures, consisting of a range of questionnaires and indexes (EuroQol (EQ-5d), Foot and Ankle Outcome Score (FAOS), Foot Function Index (FFI), Foot Health Status Questionnaire (FHSQ), and SF-36). For all but one of these studies no significant differences between the orthotic interventions were evident. A significant small effect size, indicating that the night splint resulted in a reduction in first-step pain when compared to the prefabricated arch support (ES: -0.39 (95% CI -0.76 to -0.02)) and the custom foot orthosis (ES: -0.39 (95% CI -0.74 to -0.04)), was evident from the results in Martin et al. [163]. However, no difference between the interventions was evident for pain felt during the day.

Three of the four studies from Category C provided data for the calculation of effect sizes. The non-orthotic comparators were corticosteroid injections, taping, physiotherapy, and a combination of physiotherapy and steroid injections. The greatest differences between interventions for individuals with plantar fasciitis, were seen in the studies in this category with significant large and very large effect sizes evident. Abd El Salam and Abd Elhafz [155] found superior results for a medial arch support over low dye taping for pain reduction (significant very large ES: -1.31 (95% CI -2.27 to -0.35)) but no difference was evident in the Manchester Foot Pain and Disability Index. Al-Bluwi et al. [156] provided all their participants with nonsteroidal anti-inflammatory drugs and either physiotherapy, physiotherapy and steroid injection, or an ankle brace. The ankle brace was found to be superior to the other two treatments (as evidenced by very significant large effect sizes) for both outcome measures (pain and the Short-Form McGill Pain Questionnaire). However, it should be noted that there was an unexplained unequal allocation of participants to the three treatment groups and this study was categorised as having a high risk of bias for all the entries on the Cochrane risk of bias tool.

One RCT was identified which examined cost-effectiveness. Rome et al. [165] compared accommodative and functional foot orthosis for the treatment of plantar fasciitis in the UK, with results showing that the functional orthosis was associated with a better quality-adjusted life year profile but at a higher cost to the National Health Service (NHS).

Finding from this review do not indicate a benefit to the use of orthotic interventions when compared to control conditions, however these findings are based on a single study. When comparisons were made between orthotic interventions few differences were evident. While some superior results were evident for orthotic interventions to non-orthotic interventions the quality of the research was poor. At present, there is limited research which has examined the use of orthotic interventions for the treatment of individuals with plantar fasciitis and conclusions on its effectiveness and cost-effectiveness are not possible.

#### Anterior cruciate ligament (post-surgery)

Sixteen of the identified RCTs examined the effect of knee bracing on individuals recovering from anterior cruciate ligament (ACL) surgery [173–188] with most of these studies (12 studies) comparing the provision of a brace to a control condition (Category A). Nine of the sixteen studies included suitable data to allow the calculation of effect sizes (S4 File). Within Category A data was extracted from seven articles with two of these reporting on the same study [179, 180]. The most reported outcome measure across these studies was anterior-posterior knee laxity followed by isokinetic peak muscle torque. For all the reported outcome measures no differences were evident between the control and orthotic interventions. Similarly, for the two studies for which effect sizes were calculated in Category B (comparing two types of knee braces), no differences were evident between conditions for all but one of the reported outcome measures. Results from Mayr et al. [175] indicated a superior performance for a water-filled soft brace compared to a more traditional hard brace, with the soft brace resulting in a decrease in knee extension deficit (ES: 0.85 (85% CI 0.11 to 1.59)).

Results from the identified RCTs indicate that there is no benefit to the use of knee braces on individuals recovering from ACL surgery, this finding is in line with recent research which has examined different rehabilitation interventions following ACL surgery [189, 190].

#### Diabetic foot

**Fig 9:**
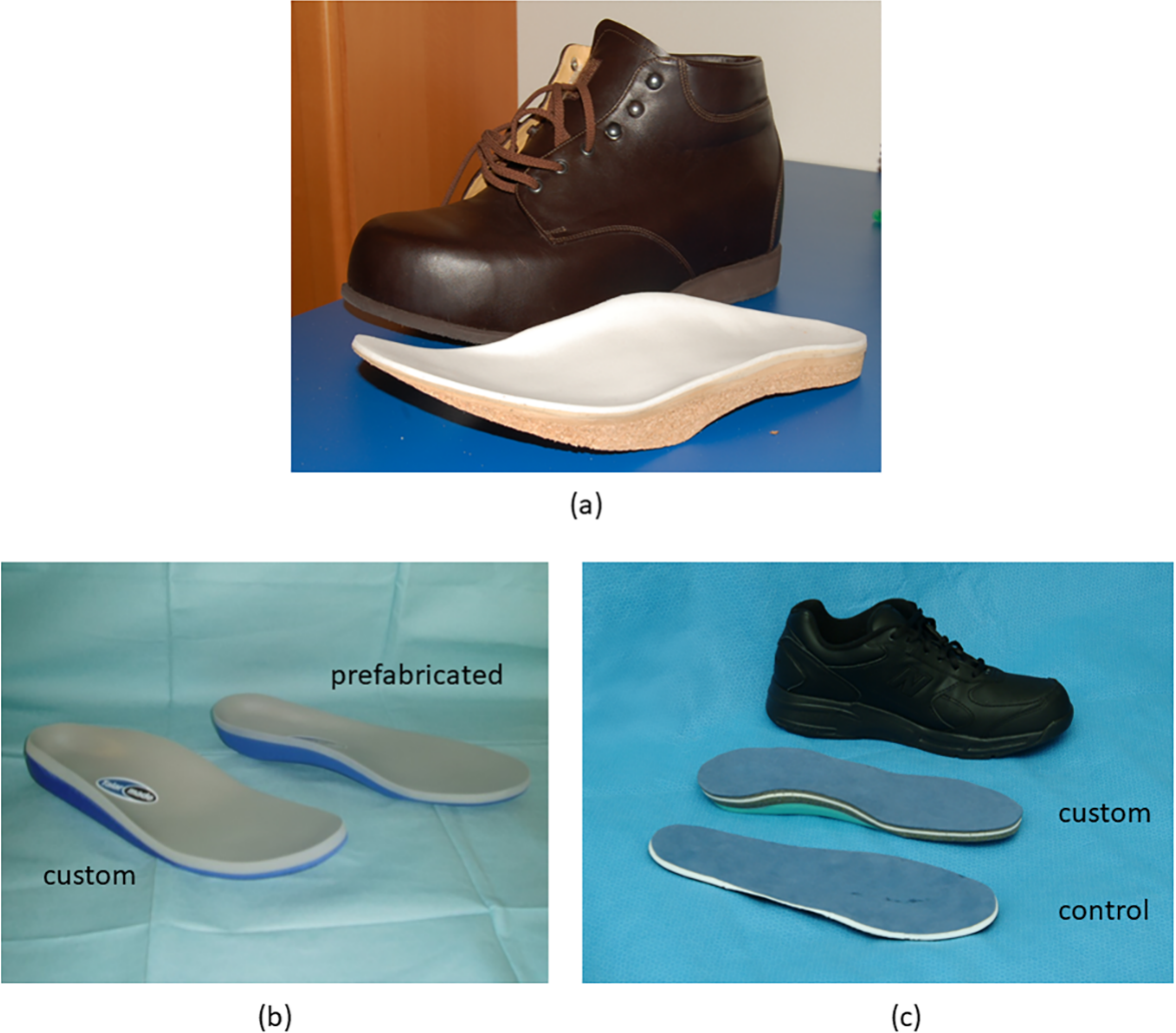
Examples, from the studies included in the systematic review, of orthotic interventions for individuals with diabetes. Custom-made shoe with custom-made insert (a) [199] (image courtesy of Dr Sicco Bus); customised and prefabricated foot orthoses (b) [200] (image courtesy of Dr Joanne Paton); control and custom foot orthoses and athletic footwear (c) [198] (image courtesy of Professor Joshua Burns).

Fifteen RCTs were identified which examined the effect of foot orthoses and/or footwear on the prevention or treatment of diabetic foot ulcers [191–205] (Fig 9). Fourteen of these fifteen studies provided data to allow calculation of effect sizes (four studies) and/or odds ratios (eleven studies). These studies examined the effectiveness of orthotic interventions for sample sizes of between 43 and 119 participants over a period of between 8 weeks and 12 months.

All four studies within Category A compared ulcer incidence or relapse in a control group to one provided with footwear and/or insoles or digital padding [202–205]. Results from three of these four studies supported the orthotic interventions with reduced ulcer incidence/relapse when compared to the control condition (odds ratios reported in S5 File) [203–205]. One study within Category A allowed calculation of the effect size for ulcer free time with a large effect size (ES: 1.59 (95% CI 0.57 to 2.61)) evident, indicating the therapeutic footwear was superior to the control condition for preventing ulcer relapse [205].

Effect sizes were calculated for two studies within Category B with these studies comparing a prefabricated to a custom orthosis [200] and a sham orthosis to a custom orthosis [198]. No differences were evident for any of the reported outcomes measures (quality of life, Bristol Foot Score, and plantar pressure measurements) from Paton et al. [200]. Similarly, for Burns et al. [198] no significant differences were evident for the quality of life measure (SF-36), Foot Health Status Questionnaire and the plantar pressure measurements.

Two studies in Category B reported on ulcer healing rates, allowing for odds ratios to be calculated. Results showed that an instant total contact cast (iTCC) was superior to a removable cast walker (RCW) (OR: 4.41 (95% CI 1.18 to 16.45))[196] and a RCW was superior to a half shoe (OR: 1.33 (95% CI 0.39 to 4.52)) [197] for ulcer healing. While Armstrong et al. [196] reported that ulcers healed for a higher number of participants in the iTCC group compared to the RCW group, a higher number of participants in the iTCC group presented with a at least one episode of periwound maceration than did those using the RCW (OR: 0.27 (95% CI 0.08 to 0.86). Three studies reported on ulcer incidence with no differences evident between the orthotic interventions in two studies [199, 202]. For one study a reduced ulcer incidence in the group which received a custom orthosis (customised by foot shape and plantar pressure) compared to a standard orthosis (OR: 0.30 (95% CI 0.11 to 0.83) was found [201].

In Category C one study compared a total contact cast to custom made footwear with no difference found between the two interventions for their ability to reduce the area of the foot ulceration over a period of 16 weeks (ES: 0.24 (-0.39 to 0.87)) [194]. Three of the five studies in this category which examined ulcer healing rates reported superior results for total contact casts (TCCs) over any of the tested orthotic interventions (RCW [192, 197], half shoe [197], shoe and insole [191]) (ORs reported in S5 File).

Miyan et al. [193] stated that differences in cost-effectiveness between the three offloading interventions (modified foot wear, modified plaster of Paris cast and boot) for diabetic ulcer healing were evident. However, they only reported on the cost of the material for their interventions and not the other elements which should be included to calculate cost-effectiveness.

When compared to a control condition, orthotic interventions showed some evidence of superior results with lower ulcer incidence/relapse rates. However, when it comes to treating active ulceration, TCCs show superior results in most the RCTs. Our findings are in line with previous research in this area [18, 206].

#### Ankle sprain

Ten RCTs were identified which examined the use of orthotic interventions in the treatment of ankle sprains [207–216], with three studies providing data on their outcome measures which allowed for the calculation of effect sizes (S4 File). The sample sizes of these studies ranged from 43 to 441 participants with follow-up periods of 3 to 9 months. Within category B, three of the five RCTs provided data with two of these studies reporting on the same study [214, 216]. The baseline values reported for the FAOS (Foot and Ankle Outcome Score) differ between these two publications. For this analysis we have taken the values from Lamb et al. [216] for effect size calculation, as this article provided standard deviation data which was not provided in Cooked et al. [214], and no data was extracted from Cooke and colleagues [214].

All three studies for which data was extracted used the FAOS as an outcome measure [212, 213, 216]. This scale normally uses an increase in score to signify an improvement (100 indicates no problems and 0 indicates extreme problems), however Best et al. [213] reported improvements with decreasing scores. No differences were evident between the interventions in any of these three studies (S4 File).

Data was extracted from two of the seven studies in Category C [212, 216]. These studies compared several different braces to a range of non-orthotic comparators (taping, tubigrip and casting). No differences were evident between the interventions for all the reported outcome measures (pain, health, FAOS subscales, SF-12, Karlsson Score and Tegner Score).

One RCT reported on cost-effectiveness; Cooke et al. [214] compared two orthotic interventions (Aircast brace and Bledsoe boot) and two non-orthotic interventions (below knee cast and tubular bandage) in the treatment of severe ankle sprain. While the below knee cast and Aircast brace were found to be more cost-effective than tubular bandage at 3 months there was no difference between interventions in the long-term.

At present, there is no indication from the available research on the use of orthotic interventions for the treatment of ankle sprains to support the use of one type of orthotic intervention over another, or to indicate that an orthotic intervention is superior to a non-orthotic intervention (tape, tubigrip or below knee cast). In line with our findings, a Cochrane review, whose search included studies published up to 2006, reported that insufficient evidence was available to determine the effectiveness of surgical and conservative treatment for acute injuries of the lateral ligament complex of the ankle [217]. In contrast to the Cochrane review, the current review did not include studies examining ligament ruptures as a part of the ankle sprain studies.

#### Cerebral palsy

Five of the eight identified RCTs [218–225] provided data which allowed for calculation of effect sizes (S4 File). One of these studies [221] did not supply sufficient information within their methods section to explain the outcome measures adequately and therefore data from this study was not extracted. These studies had samples sizes ranging from 19 and 105 and the study duration was between 3 and 12 months. A range of outcome measures were used across these studies, with Gross Motor Function Measurement and ankle dorsiflexion the only two outcomes measures reported by more than one study. One of the remaining four studies examined the provision of a knee ankle foot orthosis (KAFO) to a control condition [224] (Category A), reporting results for 19 participants over a 12 month period. No difference between the groups was calculated for the ability of the KAFO to maintain the ankle-foot dorsiflexion range of motion when compared to the control condition. The one study in this group which allowed for the calculation of an odds ratio (S5 File), Graham et al. [223], compared a control group to one provided with Botulinum Toxin A and a hip abduction brace. The control group were more likely to progress to surgery than the treatment group (23/44 progressed to surgery in the control group compared to 14/46 in the treatment group; OR: 0.4 (95% CI 0.17 to 0.95)).

Two studies compared orthotic interventions (Category B), with one study comparing placebo to postural insoles [219] and reporting on 20 participants over 3 months. The second compared the same AFO across two different usage conditions (day wear vs. day-night wear) with 105 participants over 8 weeks [220]. Across the majority of the outcome measures reported by Christovao et al. [219] no difference between the postural insoles and the placebo insoles was evident (6 minute walk test, Berg Balance Scale, centre of pressure measurement and GMFM-88). A significant very large effect sizes was evident for a reduction in the timed up and go test, ES: -1.4 (95% CI -0.05 to -2.75), indicating greater improvement for the postural insoles than the placebo insoles. From Zhao et al. [220], non-significant small effect sizes were calculated for all the extracted outcome measures, indicating no differences between day wear vs. day-night wear for the same AFO.

Within category C, one study compared Botulinum Toxin A treatment to a Johnstone pressure splint in a sample of 43 participants over a 3-month period [218]. Botulinum Toxin A treatment was superior to the Johnstone pressure splint, as indicated by a significant very large effect size for a reduction in the Modified Ashford Scale (ES: 1.41 (95% CI 0.78 to 2.04)). No differences were evident between the groups for the remaining outcome measures (knee distance, GMFM-88 and passive hip abduction).

Most of the identified RCTs which examined the paediatric population in this review investigated orthotic interventions for individuals with cerebral palsy, and although it is a chronic medical condition, orthotic interventions for adults with cerebral palsy have not been examined. From the extracted data, there were some positive findings for the combined treatment of Botulinum Toxin A and hip abduction brace compared to a control condition. There were also some positive results for postural foot orthoses, however one study found the use of Botulinum Toxin A superior to a Johnstone pressure splint. The limited number of RCTs all examined different interventions and at present there is not sufficient evidence on the effectiveness of orthotic interventions in the treatment of individuals with cerebral palsy.

#### Lateral epicondylitis

Of the eight identified RCTs [226–233], four articles (from three RCTs) included data which allowed for calculation of effect sizes (S4 File). Two studies were excluded from data extraction as they recruited and randomised participants with bilateral involvement [227, 231], which has implications for statistical analysis [154]. The studies, for which effect sizes were calculated, compared the provision of an orthotic to a non-orthotic comparator (Category C), comparing the provision of a brace to ultrasound therapy [228], laser therapy [226, 228] or physiotherapy [229, 230]. These studies had samples sizes ranging from 58 to 180 participants and the study duration was between 6 weeks and 12 months. Non-significant small effect sizes were evident for most of the reported outcomes measures (inconvenience, grip strength, pain, quality of life (EuroQol, SF-36), severity of complaints, thickness of common extensor tendon). All studies reported on a measure of pain with significant large effect sizes, equating to a greater reduction in pain, evident in two studies. Laser therapy resulted in a greater reduction in pain when compared to a brace [228] (ES: 1.04 (95% CI 0.35 to 1.73) and a brace reduced pain more than sham laser therapy [226] (ES: -0.8 (95% CI -1.45 to -0.15)).

Success rates were reported by Struijs and colleagues [230], allowing for the calculation of odds ratios. Success rates were slightly higher for physiotherapy (89%) and brace + physiotherapy (88%) groups compared to the brace group (85%) (ORs reported in S5 File).

Only one of the identified RCTs, Struijs et al. [207], examined the cost-effectiveness of the interventions they examined. They compared brace, physiotherapy, or both treatments combined in the Netherlands which no significant differences evident between the treatment interventions.

Findings on the use of bracing in the treatment of lateral epicondylitis are inconclusive with significant differences between orthotic interventions and a non-orthotic comparator evident in two of the four studies for which data was extracted. Findings are in agreement with a Cochrane review, completed in 2002, which examined orthotic interventions in the treatment of lateral epicondylitis [234]. This previous review identified five RCTs for inclusion and the authors concluded that no definitive conclusions on effectiveness of these interventions could be drawn. None of the five RCTs included in this Cochrane review were included in the present review as four of these studies were completed before 1995 and the fifth was published in the Turkish language.

#### Low back pain

Six of the eight RCTs identified which examined low back pain [235–242]) included data which allowed for the calculation of effect sizes (S4 File). Four of these studies [238–241] compared the provision of an orthotic to non-provision (Category A) with sample sizes of between 33 and 197 participants, and study durations of between 6 and 12 weeks. All four studies used assessment of pain as an outcome measure. For the two studies which examined lumbosacral orthoses [238, 241] no differences were evident for a reduction in pain with use of the orthoses. In the two studies, which examined the use of foot orthoses, significant large (ES: -0.82 (95% CI -1.51 to -0.13)) [239] and very large (ES: -1.75 (95% CI -2.79 to -0.71)) [240] effect sizes were calculated for a reduction in pain with use of the orthoses. Non-significant small and medium effect sizes were calculated for self-rated disability (EIFEL, Oswestry Disability Index and Roland-Morris Disability Questionnaire) in all four studies. The inextensible lumbosacral orthosis resulted in a greater deterioration in the Patient Specific Activity Scale than the control condition in the study by Morrisette et al. [241], ES: 0.56 (95% CI 0.01 to 1.11).

Three studies compared different orthotic interventions (Category B); comparison of flat to rocker sole shoes [237], comparison of two lumbosacral orthoses [241] and comparison of placebo to custom made foot orthoses [236]. Sample sizes ranged from 51 to 98 participants and the studies were conducted for between 2 weeks and 12 months. Mac Rae et al. [237] compared flat to rocker sole shoes with no differences evident between the groups for all reported outcome measures (Hospital Anxiety and Depression Questionnaire, Pain, Patient Specific Functional Scale, Quality of life, Roland-Morris Disability, and Tampa Scale of Kinesiophobia Questionnaire). Positive results for custom made foot orthoses compared to placebo orthoses were evident from Castro-Mendez et al. [236] with a significant very large effect size calculated for improvement in pain (ES: -1.45 (95% CI -2.21 to -0.69)) for the custom orthoses, however, no difference between groups was evident for the Oswestry Disability Index. In the comparison of two lumbosacral orthoses (inextensible and extensible) by Morrisette et al. [241], there was no difference evident between the orthoses for the reported outcome measures (pain, Fear Avoidance Beliefs Questionnaire and Oswestry Disability Index).

Finding from this review are in agreement with those of two previous Cochrane reviews which have examined the use of specific orthotic interventions, lumbar supports [243] and insoles [244], in the treatment of low back pain. Both these reviews stated that findings on the effectiveness of these interventions were inconclusive and that high quality randomised trials examining these interventions were needed. These reviews are nine and ten years old, respectively, and while several RCTs has been conducted since these reviews were completed we are no closer to a conclusive answer on the effectiveness of orthotic interventions in the treatment of low back pain.

#### Summary for orthotic interventions

From the RCTs examining orthotic interventions, it was found that across all twelve medical conditions/injuries most of the calculated effect sizes for the extracted outcome measures were small and non-significant, this was consistent across the three categories A, B and C. There were some positive results indicated for orthotic interventions in each of the medical conditions/injuries but they were also some conflicting results and due to the low methodological quality of the identified RCTs (as evidenced by the risk of bias assessment) it is not possible to make conclusions on the effectiveness orthotic interventions. There was a limited examination of orthotic interventions in paediatric populations with this population only examined in sixteen RCTs (8 cerebral palsy RCTs, 5 fracture RCTs and 3 and juvenile idiopathic arthritis RCTs).

Only 6 of the 194 RCTs which examined orthotic interventions stated that they assessed cost-effectiveness [88, 140, 165, 193, 214, 229]; four of these studies compared an orthotic intervention to a non-orthotic comparator and the remaining two studies compared orthotic interventions. None of these RCTs examined the same medical condition and all utilised different outcome measures to quantify cost-effectiveness.

### General considerations

Regarding the risk of bias assessment of the included RCTs, there was an unclear or high risk of bias across many of the domains, in particular, for the blinding of participants, personnel and outcome assessors which should be considered when evaluating the estimates of effect and odds ratios. Giving the type of interventions we are considering in this review, it is not always possible to blind participants to the intervention they are provided. In these RCTs, as is common in non-pharmacological trials, it is not always possible to use a placebo intervention within a control group. Some of the RCTs examining foot orthoses has stated using placebo orthoses, with these studies using different terminology to describe these control interventions (flat/placebo/sham/control/neutral). This methodological approach was most common in the RCTs examining the effectiveness of foot orthoses in arthritis [33, 38, 42, 49, 51, 54] but was also evident in RCTs examining foot orthotics in plantar fasciitis [161, 164], diabetes [198] and cerebral palsy [219]. There is currently some debate in the literature about the use of control interventions in trials examining orthoses, with some researchers promoting their use [245] and others recommending that participants use their own footwear as a control condition [246, 247]. Blinding outcome assessors is also a challenge in these studies; while the use of patient reported outcome measures to measure clinical service effectiveness is promoted in healthcare [248], as these participants have knowledge of their assigned intervention this can have an impact on the accuracy of their patient-reported outcomes.

From this review, it is highlighted that an extensive range of outcome measures were utilised across all clinical populations with few RCTs reporting on the same outcome measures within a specific medical condition/injury. It was also evident that studies in general did not assess if the prosthetic or orthotic interventions allowed the users to participate in the activities of daily living in which they wished to perform, which is the major consideration for the user. Potential solutions to the issue of the extensive range of outcome measures are currently being developed with a number of organisations (e.g. International Consortium for Health Outcomes Measurement (ICHOM) (http://www.ichom.org), COMET Initiative (http://www.comet-initiative.org), and Outcome Measures in Rheumatology (http://www.omeract.org/index.php)) currently working to define minimum sets of condition-specific outcome measures to enable the measurement of the value-based health care provided to patients. The development of these standard sets is not intended to prohibit the development and use of other outcome measures but to provide a set of outcome measures which researchers should use routinely.

Considering the medical conditions/injuries discussed within this review, the ICHOM have published condition specific outcome measures for hip and knee osteoarthritis [249], stroke [250], and low back pain [251] and are in the process of completing sets for diabetes and inflammatory arthritis. Since these specific outcome measure sets have been published recently, one could argue that many of the studies have not had a chance to include them in their work. However, within this review, we shall consider these papers in conjunction with the published outcome measure sets with a view to examine whether they have been utilised in the research to date.

For hip and knee osteoarthritis [249] the recommended outcome measure for pain was the numeric pain rating scale, the majority of the RCTs in this review utilised visual analogue scales for pain measurement. While both numerical and visual scales for pain assessment have been validated some research has shown that numerical rating is superior to verbal or visual scales [252]. It was recommended to use the Knee Injury and Osteoarthritis Outcome Score—Physical Function Short form (KOOS-PS) [253], and the Hip Disability and Osteoarthritis Outcome Score—Physical Function Short form (HOOS-PS)) [254] to access physical functioning, and the EQ-5D-3L [255] or the SF-12 [256] for measuring health related quality of life. Only two of the identified RCTs examining orthotic interventions in knee osteoarthritis in this review utilised a least one of these outcome measures [58, 68].

For stroke [250], the recommended outcome measures for patient reported health status were the PROMIS SF v1.1 Global Health [257] and the simplified modified Rankin Scale questionnaire [258]; neither of these measures were utilised in any of the RCTs examining orthotic interventions in participants post stroke in this review. The standard set for low back pain [251] recommends the use of the Oswestry Disability Index [259] to assess disability; three of the RCTs in this review utilised this index [236, 239, 241]. The numeric pain rating scale for pain measurement was recommend with the authors discussing the limitations to both visual and numerical pain scale. While the visual scale allows a more specific response the numerical scale is considered easier to use as it can be performed verbally and does not require exact size calibration. For the assessment of health related quality of life the EuroQol-5D (EQ-5D) [255] was recommended; only one study in this review utilised this outcome measure [237]. It should be noted that as these standard sets have been developed recently and are derived from expert consensus rather than high levels of evidence they require validation. While these standard sets, discussed above, have the advantage of being population specific there may also be a need to define core sets of outcome measures to specifically examine the effectiveness of prosthetic and orthotic interventions [260–262].

In addition to the extensive range of outcome measures reported in the identified RCTs there were inconsistencies across studies in the reporting of the primary outcomes measures. Across all medical conditions there were many RCTs which did not state the primary outcome measure(s) for their study. This finding is not unexpected as previous research has identified this is an issues across all medical research, with only 53% of 614 PubMed-Indexed RCTs published in 2006 found to specify their primary outcome [263]. In the RCTs which did specify a primary outcome measure in the present review it was noted that pain was the most utilised within RCTs examining arthritis and plantar fasciitis, with a wide variety of outcome measures chosen as primary outcomes in the other medical conditions/injuries. For example, plantar pressure was measured in two studies which examined the effectiveness of foot orthoses in individuals with diabetes; Paton et al. [200] identified the plantar pressure outcome measures as primary outcome measures while Burns et al. [198] identified these measures as secondary outcome measures.

There were RCTs identified which utilised the same outcome measures, however, comparisons across some of these studies were complicated by how the results were presented. For example, higher scores on the WOMAC indicate worse pain, stiffness, and functional limitations, yet van Raaij et al. [55] reported improvement with an increase in the WOMAC score and while the majority of studies used a reduction in pain scores to indicate improvement Rafiaee and Karimi [48] used an increased value to indicate improvement.

While this review found that a limited number of RCTs have been completed which examined prosthetic and orthotic interventions for most medical conditions/injuries there was a significant body of studies which was not included as it did not meet the inclusion criteria. These studies consisted of cross sectional, cross over and prospective uncontrolled trials. Additionally, many systematic and narrative reviews were identified during the current search. As was found with the identified RCTs in this review, the clear majority of these reviews were on provision of orthotics with only two reviews examining prosthetic interventions. Both these prosthetic intervention reviews examined lower limb prosthetics. Many of the reviews on orthotic interventions for adults examined their use in the treatment or rehabilitation of fractures. Most of these studies concluded that sufficient evidence on the examined interventions is currently not available and discussed the lack of high quality RCTs and the urgent need for research in this area.

While at present the scientific literature does not provide sufficient high quality prospective studies to allow strong conclusions on the effectiveness or cost-effectiveness of prosthetic and orthotic interventions there are a number of reports from various organisations in the UK and US which discuss the positive impact of orthotic [264–267] and prosthetic [268, 269] service provision.

As discussed above there are many methodological issues within this area of research (use of placebo interventions / blinding participants, defining standard sets of outcome measures, identification of primary outcome measures) which need to be addressed to strengthen the quality of future research, which would then allow conclusive decisions to be made on the effectiveness and cost-effectiveness of these interventions.

## Limitations

Most of the RCTs examined in this review assessed their outcome measures at multiple time points during their intervention periods, however we limited our analysis to comparing the outcome measures at baseline and the final assessment. This may have resulted in missing incidences where significant differences between were evident at specific follow up time points. The ability to extract data from RCTs examining acute injuries was limited as effect size calculation requires baseline data. For this reason, it was only possible to extract data from a small number of RCTs examining the effectiveness of orthotic interventions in the treatment of fractures (ten of twenty-six studies) and ankle sprains (three of ten studies).

## Conclusions

There is a lack of RCTs examining prosthetic interventions, with only two studies completed to date which have examined effectiveness and neither assessed the cost-effectiveness of the interventions. Regarding orthotic interventions, this review identified the wide range of medical conditions/injuries where these interventions are utilised, however, there is a lack of evidence from the available research to draw conclusions on the effectiveness of these interventions.

## Supporting information

S1 File PROSPERO protocol. (PDF)

S2 File MEDLINE search strategy search terms. (DOCX)

S3 File Effect size and odds ratio equations. (DOCX)

S4 File Calculated effect sizes for outcome measures of each included study. (XLSX)

S5 File Calculated odds ratio for outcome measures of each included study. (XLSX)

S6 File Risk of bias figures. (DOCX)

S1 Table PRISMA checklist. (DOC)
