# Supplementary material for: A systematic review of randomised controlled trials assessing effectiveness of prosthetic and orthotic interventions
Source: PLoS One. 2018 Mar 14;13(3):e0192094. doi: 10.1371/journal.pone.0192094 (PMC5851539; doi:10.1371/journal.pone.0192094)
Supplement: S1 File — (PDF) [file pone.0192094.s001.pdf]

Effectiveness and cost effectiveness of prosthetics and orthotics services/interventions: a systematic review

Aoife Healy, Nachiappan Chockalingam, Sybil Farmer, Nicola Eddison, Anand Pandyan

Citation

Aoife Healy, Nachiappan Chockalingam, Sybil Farmer, Nicola Eddison, Anand Pandyan. Effectiveness and cost effectiveness of prosthetics and orthotics services/interventions: a systematic review. PROSPERO 2016:CRD42016025994 Available from
http://www.crd.york.ac.uk/PROSPERO_REBRANDING/display_record.asp?ID=CRD42016025994

Review question(s)

What is the effectiveness of prosthetics and orthotics services/interventions?

What is the cost-effectiveness of prosthetics and orthotics services/interventions?

Searches

Search strategies will be developed using medical subject headings (MeSH) and text words related to orthotics and prosthetics. Databases we will use in the search include Web of Science, MEDLINE, PubMed, CINAHL Plus, EMBASE, PsycINFO, SCOPUS, REHABDATA, NIHR and the CEA Registry. PROSPERO will be searched for ongoing or recently completed systematic reviews. We will also search selected business, economic, education and engineering databases such as Business Source Complete, Education Research Complete, ERIC, IEEE.

Types of study to be included

We will include randomised control trials (RCTs), controlled (non-randomised) clinical trials, controlled before-after studies, prospective and retrospective comparative cohort studies and case-control studies. Both qualitative and quantitative studies will be sought. Case series and case report studies will be excluded. We will include articles reported in the English language. A list of possible relevant titles in other languages will be provided as an appendix.

Condition or domain being studied

According to the World Report on Disability, there are more than 1000 million people with disability worldwide, about 15% of the global population. Of this number, between 110 million and 190 million adults experience significant difficulties in functioning. It is estimated that some 93 million children – or one in 20 of those under 15 years of age – live with a moderate or severe disability. The majority of this population would benefit from prosthetics and orthotics services, if available within a country. The prevalence of disability is rising because of aging populations and the global increase in chronic disease conditions. There is no definite data available at this stage but it is estimated that at least in excess of 100 million people (1.5 % of the world’s population) are in need of prosthetic/orthotic services.

Participants/ population

People with physical impairments or limb loss or functional limitations or deformities in limb or spine or head. No restriction will be set on age, gender or the severity of the condition.

Intervention(s), exposure(s)

Provision of prosthetics/orthotics services/interventions.

Comparator(s)/ control

Non-provision of prosthetics/orthotics services/interventions or provision of alternative assistive products (such as crutches, walkers, sitting board with castors, wheelchairs, and tricycles).

Studies which compare different prosthetics/orthotics services/interventions.

Studies which compare orthotics services/interventions to other treatments/interventions (e.g. surgery)

Context
Services/interventions delivered in any setting will be considered.

Outcome(s)
Primary outcomes
The review is interested in the effect of prosthetic and orthotic services/interventions on an individual's function and the cost effectiveness of these services. Relevant outcomes measures assessing these areas will be eligible.

Secondary outcomes
Relevant outcomes measures assessing the effect of prosthetic and orthotic services/interventions on an individual's physical activity and societal participation.

Data extraction, (selection and coding)
Two reviewers will independently screen the titles and abstracts yielded by the search against the eligibility criteria, to reduce the likelihood of errors. We will obtain full reports for all titles that appear to meet the inclusion criteria or where there is any uncertainty. Any dispute regarding eligibility between reviewers will be resolved through discussion.

Following the initial screening, both reviewers will then independently screen the full text articles to assess whether these meet the eligibility criteria. Any disagreements regarding study eligibility between reviewers will be resolved through discussion.

A 'Preferred Reporting Items of Systematic reviews and Meta-Analyses' (PRISMA) flowchart will be completed to demonstrate the number of citations retrieved and the number excluded at each stage.

A standardized data extraction form will be developed based on the data collection forms created by the Cochrane Collaboration. Raw data extraction will include study design, participant characteristics (number of participants, age, sex) and description of experimental and comparison interventions, co-interventions, adverse effects, duration of follow-up, outcomes assessed and results. Data will be extracted by one reviewer and checked by another reviewer.

Risk of bias (quality) assessment
The methodological quality of the included studies will be assessed by two reviewers independently with any disagreements resolved through discussion. We will assess the risk of bias in included studies using the risk of bias tool described in the Cochrane Handbook for Systematic Reviews of Interventions.

Strategy for data synthesis
Outcome measures from individual trials will be combined through meta-analysis where possible. If a meta-analysis is not possible, the results from clinically comparable trials will be described qualitatively in the text.

Analysis of subgroups or subsets
If possible data will be sub grouped based on medical conditions and age (adults and children).

Dissemination plans
In addition to providing a report to the the International Society for Prosthetics and Orthotics and the World Health Organization, we will also disseminate our findings as widely as possible. This will include presenting at various member society conferences and publishing in peer reviewed journals.

Contact details for further information
Dr Healy

Science Centre (R009),

Staffordshire University,

Leek Road,

Stoke On Trent,

ST4 2DF

a.healy@staffs.ac.uk

Organisational affiliation of the review
Staffordshire University

www.staffs.ac.uk

Review team
Dr Aoife Healy, Staffordshire University, Stoke On Trent, UK
Professor Nachiappan Chockalingam, Staffordshire University, Stoke On Trent, UK
Dr Sybil Farmer, Staffordshire University, Stoke On Trent, UK
Ms Nicola Eddison, Royal Wolverhampton NHS Trust, Wolverhampton, UK
Professor Anand Pandyan, Keele University, Keele, UK

Collaborators
Dr Zulfiqarali Abbas, Abbas Medical Centre, Dar es Salaam, Tanzania
Dr Anil Kumar Jain, Dr. P.K. Sethi Rehabilitation Centre, Jaipur, India
Professor Stefano Negrini, University of Brescia, Brescia, Italy
Dr Elizabeth Hardin, Cleveland FES Center, Cleveland, USA
Professor Isabel Sacco, University of Sao Paulo, Sao Paulo, Brazil
Professor Saeed Zahedi, Blatchford Products Ltd., Basingstoke, UK
Dr Sarah Curran, Cardiff Metropolitan University, Cardiff, UK
Dr Géza Kogler, Georgia Institute of Technology, Atlanta, USA
Professor Anthony Ward, Staffordshire & Stoke on Trent Partnership Trust, Stoke On Trent, UK

Anticipated or actual start date
07 September 2015

Anticipated completion date
07 September 2016

Funding sources/sponsors
International Society for Prosthetics and Orthotics (ISPO)

(Sub-grant from ISPO’s Collaborative Agreement with USAID Rehabilitation of physically disabled people in developing countries – USAID cooperative agreement DFD-A-00-08-00309-00.)

Conflicts of interest
None known

Language
English

Country
England, Wales, Brazil, India, Italy, Tanzania, United States of America

Subject index terms status
Subject indexing assigned by CRD

Subject index terms
Cost-Benefit Analysis; Humans; Orthotic Devices

Stage of review
Ongoing

Date of registration in PROSPERO
05 March 2016

Date of publication of this revision
05 March 2016

Stage of review at time of this submission	Started	Completed
Preliminary searches	No	Yes
Piloting of the study selection process	No	Yes
Formal screening of search results against eligibility criteria	No	Yes
Data extraction	Yes	No
Risk of bias (quality) assessment	Yes	No
Data analysis	No	No

PROSPERO
International prospective register of systematic reviews
The information in this record has been provided by the named contact for this review. CRD has accepted this information in good faith and registered the review in PROSPERO. CRD bears no responsibility or liability for the content of this registration record, any associated files or external websites.
