# Supplementary material for: A systematic review of randomised controlled trials assessing effectiveness of prosthetic and orthotic interventions
Source: PLoS One. 2018 Mar 14;13(3):e0192094. doi: 10.1371/journal.pone.0192094 (PMC5851539; doi:10.1371/journal.pone.0192094)
Supplement: S2 File — (DOCX) [file pone.0192094.s002.docx]

**S1 File.**

**MEDLINE Search Strategy Search Terms.**

1. Medline; ORTHOTIC DEVICES/;

2. Medline; BRACES/;

3. Medline; FOOT ORTHOSES/;

4. Medline; ARTIFICIAL LIMBS/;

5. Medline; orthoses.af;

6. Medline; orthosis.af;

7. Medline; orthotic*.af;

8. Medline; brace*.af;

9. Medline; "arch support*".af;

10. Medline; insole*.af;

11. Medline; "shoe insert*".af;

12. Medline; "footwear insert*".af;

13. Medline; "boot insert*".af;

14. Medline; (prescript* ADJ2 (shoe* OR footwear OR boot*)).af;

15. Medline; ("extra depth" ADJ2 (shoe* OR footwear OR boot*)).af;

16. Medline; ((orthopedic OR orthopaedic) ADJ2 (shoe* OR footwear OR boot*)).af;

17. Medline; (therapeutic* ADJ2 (shoe* OR footwear OR boot*)).af;

18. Medline; ("custom made" ADJ2 (shoe* OR footwear OR boot*)).af;

19. Medline; (("post operative" OR postoperative) ADJ2 (shoe* OR footwear OR boot*)).af;

20. Medline; (stock ADJ2 (shoe* OR footwear OR boot*)).af;

21. Medline; (rocker ADJ2 (shoe* OR footwear OR boot*)).af;

22. Medline; (bespoke ADJ2 (shoe* OR footwear OR boot*)).af;

23. Medline; "healing sandal*".af;

24. Medline; "cast walker*".af;

25. Medline; "leg caliper*".af;

26. Medline; "walking caliper*".af;

27. Medline; (prosthes* ADJ2 (arm* OR leg* OR limb*)).af;

28. Medline; (prosthetic* ADJ2 (arm* OR leg* OR limb*)).af;

29. Medline; "artificial limb*".af;

30. Medline; "artificial leg*".af;

31. Medline; "artificial arm*".af;

32. Medline; SPLINTS/;

33. Medline; splint*.af;

34. Medline; SELF-HELP DEVICES/;

35. Medline; ((assistive OR "self help") ADJ2 (tech* OR device*)).af;

36. Medline; 1 OR 2 OR 3 OR 4 OR 5 OR 6 OR 7 OR 8 OR 9 OR 10 OR 11 OR 12 OR 13 OR 14 OR 15 OR 16 OR 17 OR 18 OR 19 OR 20 OR 21 OR 22 OR 23 OR 24 OR 25 OR 26 OR 27 OR 28 OR 29 OR 30 OR 31 OR 32 OR 33 OR 34 OR 35;

37. Medline; "randomized controlled trial".pt;

38. Medline; (random* OR placebo* OR "single blind*" OR "double blind*" OR "triple blind*").ti,ab;

39. Medline; ((comment OR editorial OR "meta-analysis" OR "practice guideline" OR review OR letter OR "journal correspondence") NOT "randomized controlled trial").pt;

40. Medline; ("random sampl*" OR "random digit*" OR "random effect*" OR "random survey*" OR "random aggression") NOT "randomized controlled trial*".pt;

41. Medline; (37 OR 38) NOT (39 OR 40);

42. Medline; exp COHORT STUDIES/;

43. Medline; cohort*.af;

44. Medline; "controlled clinical trial".pt;

45. Medline; 42 OR 43 OR 44;

46. Medline; exp CASE-CONTROL STUDIES/;

47. Medline; (case* ADJ5 control*).af;

48. Medline; 46 OR 47;

49. Medline; review.pt;

50. Medline; (review OR "meta analys*" OR MEDLINE).ti,ab;

51. Medline; 49 OR 50;

52. Medline; DELPHI TECHNIQUE/;

53. Medline; delphi.ti,ab;

54. Medline; 52 OR 53;

55. Medline; 41 OR 45 OR 48 OR 51 OR 54;

56. Medline; "case reports".pt;

57. Medline; 55 NOT 56;

58. Medline; 36 AND 57;

59. Medline; 58 [Limit to: Publication Year 1995-2015 and Humans];
