# Supplementary material for: A systematic review of randomised controlled trials assessing effectiveness of prosthetic and orthotic interventions
Source: PLoS One. 2018 Mar 14;13(3):e0192094. doi: 10.1371/journal.pone.0192094 (PMC5851539; doi:10.1371/journal.pone.0192094)
Supplement: S3 File — (DOCX) [file pone.0192094.s003.docx]

**S2 File.**

**Effect Size Equations.**

**Effect size =** $\frac{\bar{X_{G2}}-\bar{X_{G1}}}{\sqrt{\frac{(N_{G2}-1)\delta_{G2}^{2}+(N_{G2}-1)\delta_{G1}^{2}}{N_{G2}+N_{G2}-2}}}$ **Equation 1**

**Standard Error of Effect size =** $\sqrt{\frac{N_{G2}+N_{G1}}{N_{G2}N_{G1}}+\frac{{(\bar{X_{G2}}-\bar{X_{G1}})}^{2}}{2(N_{G2}+N_{G1)}}}$ **Equation 2**

Abbreviations: G1: Group 1; G2: Group 2; X: Mean outcome; N: Number of participants; δ: Standard error.

**Odds Ratio Equation.**

**Odds ratio =** $\frac{Group 2 odds}{Group 1 odds}$ **Equation 3**

**Group 2 odds =** $\frac{Number who did not benefit}{Number who benefitted}$ **Equation 4**

**Group 1 odds =** $\frac{Number who did not benefit}{Number who benefitted}$ **Equation 5**
