# Supplementary material for: A systematic review of randomised controlled trials assessing effectiveness of prosthetic and orthotic interventions
Source: PLoS One. 2018 Mar 14;13(3):e0192094. doi: 10.1371/journal.pone.0192094 (PMC5851539; doi:10.1371/journal.pone.0192094)
Supplement: S6 File — (DOCX) [file pone.0192094.s006.docx]

**S6 File. Risk of bias figures**

Fig A: Risk of bias graph for all prosthetic intervention RCTs: review authors’ judgements about each risk of bias item presented as percentages across all included studies.

Fig B: Risk of bias graph for all orthotic intervention RCTs: review authors’ judgements about each risk of bias item presented as percentages across all included studies.

Fig C: Osteoarthritis risk of bias graph: review authors’ judgements about each risk of bias item presented as percentages across all included studies.

Fig D: Fracture risk of bias graph: review authors’ judgements about each risk of bias item presented as percentages across all included studies.

Fig E: Stroke risk of bias graph: review authors’ judgements about each risk of bias item presented as percentages across all included studies.

Fig F: Carpal tunnel syndrome risk of bias graph: review authors’ judgements about each risk of bias item presented as percentages across all included studies.

Fig G: Plantar fasciitis risk of bias graph: review authors’ judgements about each risk of bias item presented as percentages across all included studies.

Fig H: Anterior cruciate ligament risk of bias graph: review authors’ judgements about each risk of bias item presented as percentages across all included studies.


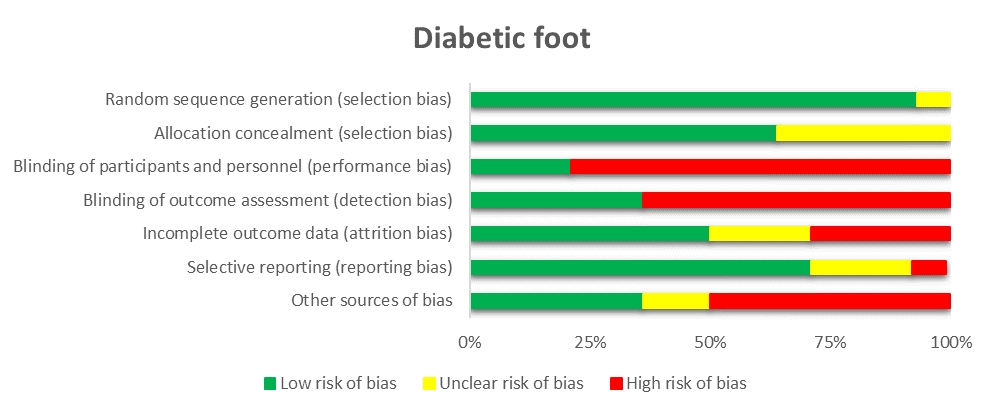


Fig I: Diabetic foot risk of bias graph: review authors’ judgements about each risk of bias item presented as percentages across all included studies.

Fig J: Osteoarthritis risk of bias graph: review authors’ judgements about each risk of bias item presented as percentages across all included studies.

Fig K: Ankle sprain risk of bias graph: review authors’ judgements about each risk of bias item presented as percentages across all included studies.

Fig L: Cerebral palsy risk of bias graph: review authors’ judgements about each risk of bias item presented as percentages across all included studies.

Fig M: Lateral epicondylitis risk of bias graph: review authors’ judgements about each risk of bias item presented as percentages across all included studies.

Fig N: Low back pain risk of bias graph: review authors’ judgements about each risk of bias item presented as percentages across all included studies.
